# MicroRNA-26a/b have protective roles in oral lichen planus

**DOI:** 10.1038/s41419-019-2207-8

**Published:** 2020-01-06

**Authors:** Jie Du, Ruifang Gao, Yimei Wang, Tivoli Nguyen, Fang Yang, Yongyan Shi, Tianjing Liu, Wang Liao, Ran Li, Fang Zhang, Xuejun Ge, Bin Zhao

**Affiliations:** 1 Department of Oral Medicine, Shanxi Medical University School and Hospital of Stomatology, Taiyuan, Shanxi China; 2 Institute of Biomedical Research, Shanxi Medical University, Taiyuan, Shanxi China; 3 Department of Endodontics, Shanxi Medical University School and Hospital of Stomatology, Taiyuan, Shanxi China; 4 Division of Biological Sciences, Department of Medicine, The University of Chicago, Chicago, IL USA; 5 Department of Periodontics, Shanxi Medical University School and Hospital of Stomatology, Taiyuan, Shanxi China; 6 Department of Pediatrics, Shengjing Hospital of China Medical University, Shenyang, Liaoning China; 7 Department of Pediatric Orthopedics, Shengjing Hospital of China Medical University, Shenyang, Liaoning China; 8 Department of Cardiology, Hainan General Hospital, Hainan Clinical Medicine Research Institution, Haikou, China; 9 Department of prosthodontics, Shanxi Medical University School and Hospital of Stomatology, Taiyuan, Shanxi China

**Keywords:** miRNAs, Inflammatory diseases

## Abstract

Oral lichen planus (OLP) is a kind of oral epithelial disorder featured with keratinocyte apoptosis and inflammatory reaction. The pathogenesis of OLP remains an enigma. Herein, we showed that the levels of miR-26a/b were robustly down-regulated in oral mucosal biopsies, serum and saliva in OLP patients compared with healthy control. Moreover, we found the binding sites of vitamin D receptor (VDR) in the promoter regions of miR-26a/b genes and proved that the induction of miR-26a/b was VDR dependent. The reduction of miR-26a/b expression was also detected in the oral epithelium of vitamin D deficient or *VDR* knockout mice. miR-26a/b inhibitors enhanced apoptosis and Type 1T helper (Th1) cells-related cytokines production in oral keratinocytes, whereas miR-26a/b mimics were protective. Mechanistically, we analyzed miRNA target genes and confirmed that miR-26a/b blocked apoptosis by directly targeting Protein Kinase C δ (PKCδ) which promotes cellular apoptotic processes. Meanwhile, miR-26a/b suppressed Th1-related cytokines secretion through targeting cluster of the differentiation 38 (CD38). In accordant with miR-26a/b decreases, PKCδ and CD38 levels were highly elevated in OLP patients’ samples. Taken together, our present investigations suggest that vitamin D/VDR-induced miR-26a/b take protective functions in OLP via both inhibiting apoptosis and impeding inflammatory response in oral keratinocytes.

## Introduction

Oral lichen planus (OLP) is considered to be a chronic mucosal disease and becoming increasingly common in dental clinics across the whole globe^1^. The prevalence of OLP has been estimated to be ~2% and this number worldwide is growing^2^. Although most studies and investigators tend to classify OLP to be an inflammatory disorder, the pathogenesis of it remains unclear for a long time^1^. Several contributing factors and triggers, such as systemic or local hypersensitivity, mental stress, microorganism infection and autoimmune response to antigen, have been involved in the beginning or development of OLP^3^. Clinically, the majority of OLP patients experience oral discomfort, even in the process of spicy or acidic foods intake^4^. Only a small group of OLP patients (about 15%) accompany with cutaneous lesions and around 0.4–5% patients with OLP have a risk of developing oral carcinoma^5^. In histopathology, the main characteristics of diseased tissues of OLP include T lymphocytes-infiltrated band in lamina propria and cytoid bodies in the epithelial layer, which represent the presence of inflammatory response and apoptosis, respectively^1,6,7^. So far efforts to manage the patients suffering OLP have been emphasized, however the therapy is only directed at controlling clinical symptoms, but not curative^1^. Given that, investigations concerning etiologic exploration and permanent cure for OLP are required.

MicroRNAs are a group of 21–25 nucleotides, noncoding RNAs that function as suppressor of gene expression upon promoting mRNA degradation of target gene posttranscriptionally^8,9^. They bind to the 3′ untranslated region (3′UTR) of mRNA directly, resulting in translation repression of downstream target genes^9^. MicroRNAs have been reported to play critical roles not only in cellular homeostasis and biological processes, but also in the development of various diseases in broad pathological conditions^8^. Among the thousands of discovered miRNAs, some of them have been demonstrated to correlate with the course of OLP^10,11^. In 2019, we reported NF-κB pathway-induced miR-802 targets B-cell lymphoma 2 (Bcl-2) mRNA to aggravate keratinocyte apoptosis in OLP^6^, establishing a correlation between miRNAs and the onset or development of this disorder. Moreover, another interesting finding noted miR-155 and miR-19a regulate Th1/Th2 balance to control inflammatory reaction in OLP^7^. Thus, exploitation of miRNA network in oral keratinocytes may offer a special window into the better explanation of OLP causes.

The miR-26 family, which contains miR-26a and miR-26b, plays a crucial role in a number of biological or pathological processes^12^. The genomic loci of miR-26a/b are located in the introns of their host genes, which code proteins of carboxy-terminal domain RNA polymerase II polypeptide A small phosphatase (CTDSP) family^13^. miR26a/b have been mainly reported to act as a tumor suppressor in several kinds of cancers by targeting genes related with cell cycle and proliferation^14–16^. In addition, recent studies showed that miR-26 suppresses TNFα, IL-6 and NF-κB pathway in bronchial epithelial cells^17^, indicating its roles in regulating inflammatory reaction. Although the functions of miR-26a/b in cancer cells is well investigated, its significance in oral keratinocytes is still elusive.

In this study, we confirmed miR-26a and miR-26b are down-regulated in OLP patient. The decreases of miR-26a/b are due to vitamin D receptor (VDR) reduction, and a positive correlation between them has been verified. In mechanism, we found that miR-26a/b target PKCδ and CD38 genes to inhibit apoptosis and Th1-related cytokines production in oral keratinocytes, respectively. Together, our findings depict that miR-26a/b take protective actions in OLP and provide a potential therapeutic target for this disease.

## Results

### miR-26a/b are down-regulated in OLP disease

**Fig. 1:**
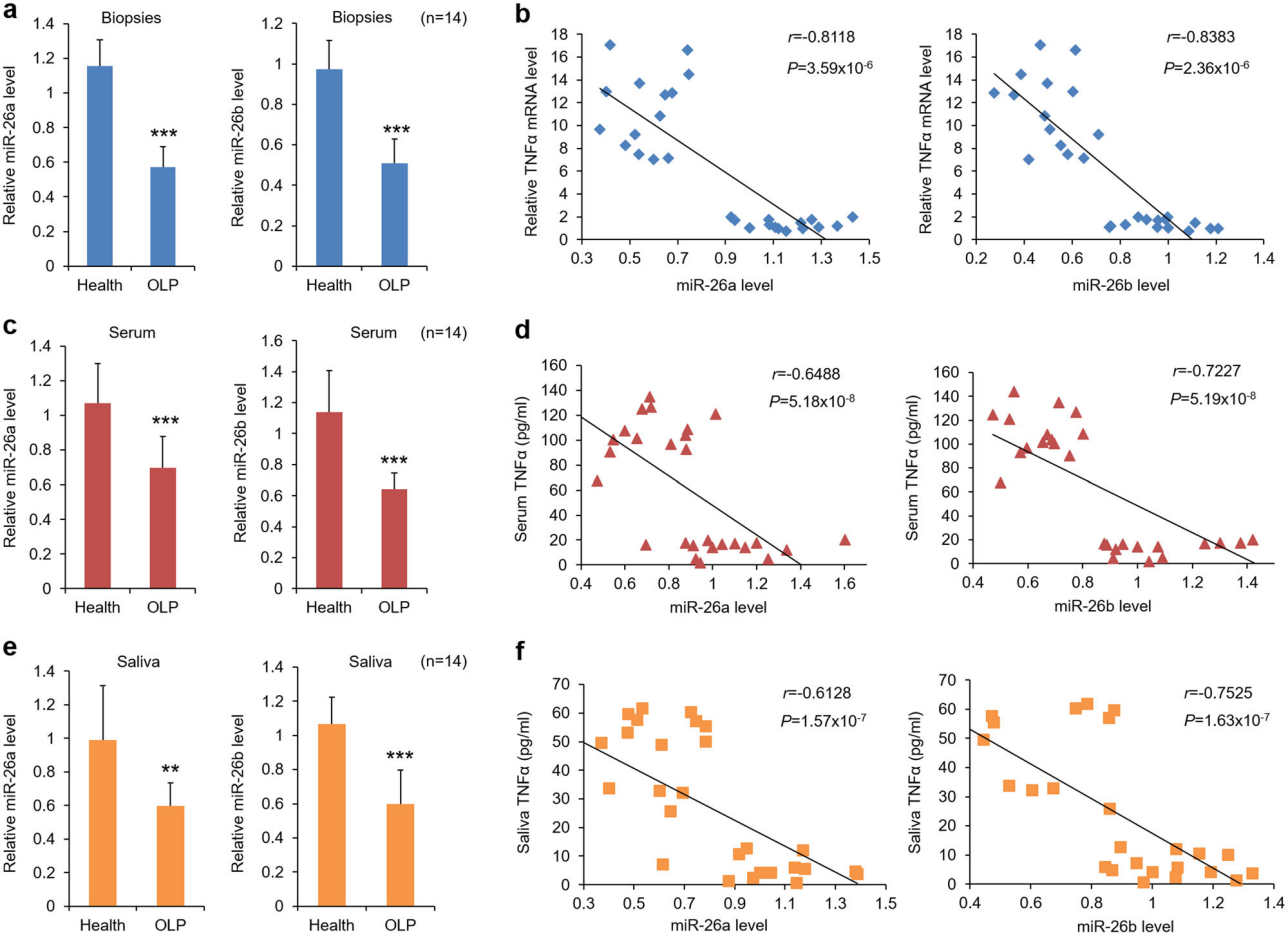
miR-26a/b are decreased in OLP patients. **a** miR-26a/b levels in human oral epithelial samples determined by real-time PCR. **b** Correlation of fold change between TNFα and miR-26a/b in oral epitheliums from OLP patients (*r* = −0.8118, *P* = 3.59 × 10^−6^, Spearman’s correlation test for miR-26a; *r* = -0.8383, *P* = 2.36 × 10^-6^, Spearman’s correlation test for miR-26b). **c** miR-26a/b levels in serum of participants measured by real-time PCR. **d** Correlation between TNFα concentrations and miR-26a/b levels in serum of OLP patients (*r* = −0.6488, *P* = 5.18 × 10^-8^, Spearman’s correlation test for miR-26a; *r* = −0.7227, *P* = 5.19 × 10^-8^, Spearman’s correlation test for miR-26b). **e** qPCR analysis of OLP saliva showing 40% decreases of miR-26a/b versus healthy controls. **f** Correlation between TNFα status and miR-26a/b expression in saliva derived from OLP patients (*r* = −0.6128, *P* = 1.57 × 10^−7^, Spearman’s correlation test for miR-26a; *r* = −0.7525, *P* = 1.63 × 10^−7^, Spearman’s correlation test for miR-26b). **P < 0.01, ***P < 0.001 vs. corresponding healthy controls; *n* = 14 each group.

As miR26a/b have important functions in regulating disease progression, we tested the status of them in the epithelial layer of OLP samples at first. As shown in Fig. 1, the levels of miR-26a and miR-26b were significantly lower in epithelium of OLP biopsies than those in healthy controls (Fig. 1a). OLP is an inflammatory disorder and indicated by enhanced expression of cytokines^1,18^. To evaluate the clinical relevance of miR-26a/b and OLP, we tested a key cytokine, TNFα, in specimens and a negative correlation was observed between them (Supplementary Fig. 1a, b). Consistently, similar results were found in serum and saliva samples derived from participants (Fig. 1c–f and Supplementary Fig. 1b, c). Since bacterial infection and autoimmune response are responsible for OLP initiation^1,19^, we established two cell models in human oral keratinocytes (HOKs) by means of LPS or activated CD4^+^ T cells’ secretion treatment, as described previously^6^, to mimic the microenvironment of OLP in vitro. As expected, TNFα levels were largely elevated while miR26a/b showed time course-dependent decreases in the presence of LPS or activated CD4^+^ T cells secretion (Supplementary Fig. 1d–g), and significant negative correlations were also found between them (Supplementary Fig. 1h–k). To date there is no sophisticated animal models for mimicking OLP, thus we were limited to test the expression of miR26 in mouse models.

### Vitamin D/VDR signaling mediates miR-26a/b expression in oral keratinocytes

**Fig. 2:**
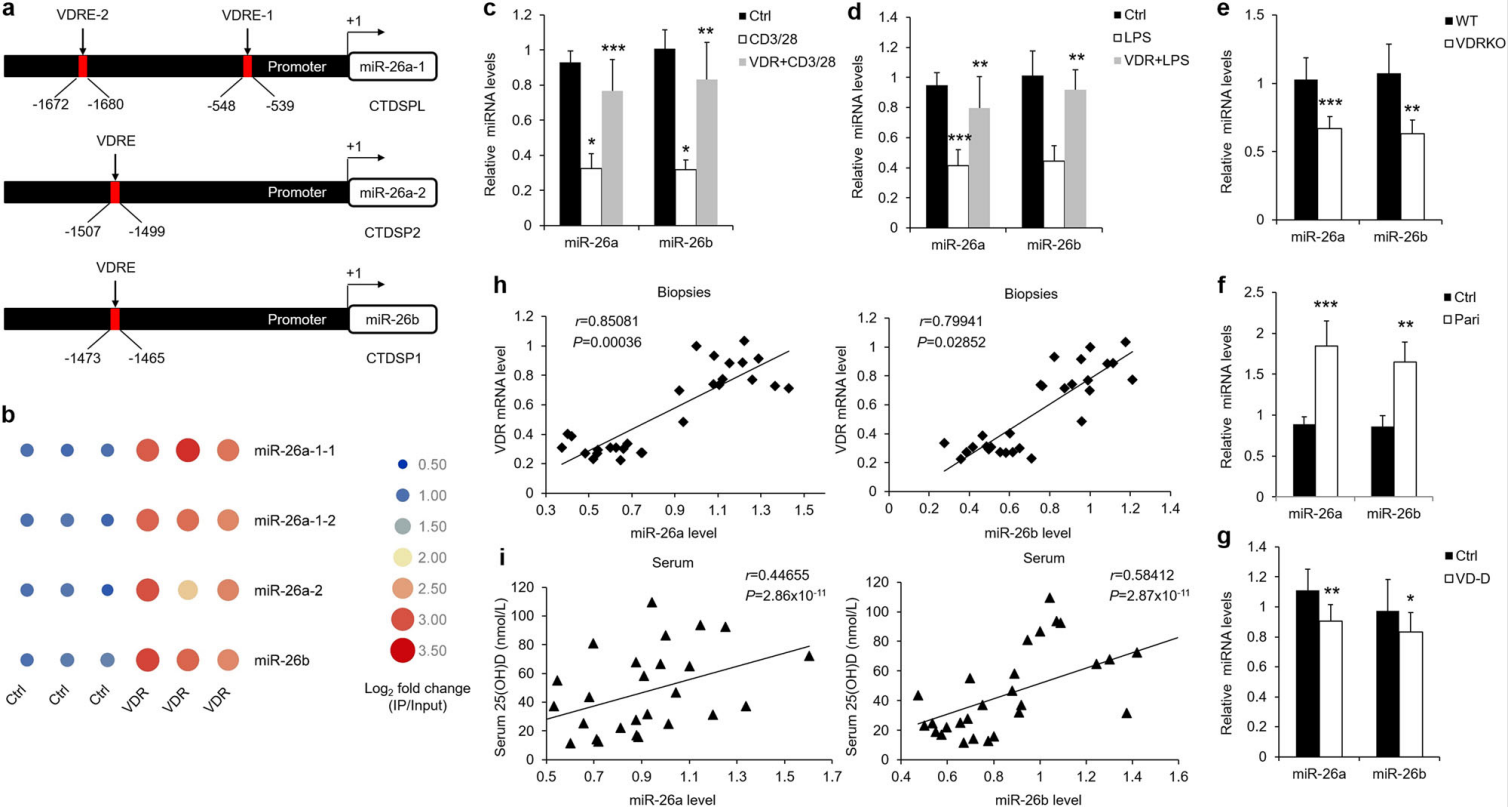
VDR induces miR-26a/b by binding with VDRE in HOKs. **a** Schematic illustration of VDR binding sites in the promoter regions of miR-26a/b genes. **b** ChIP analysis showing the increases of miR-26a/b levels after 36-hour VDR plasmids transfection in HOKs, bar indicates log_2_ fold change, *n* = 3. qPCR analysis of miR26-a/b expression in HOKs challenged by activated CD4^+^ T cells (**c**) or LPS (**d**) with or without VDR plasmids, *n* = 3. Differential expression of miR-26a/b in oral keratinocytes of VDRKO (**e**), paricalcitol-treated (**f**), or vitamin D-deficient (**g**) mice, *n* = 5. Paricalcitol is an analog of vitamin D. **h** Correlation of fold change in OLP biopsies between VDR and miR-26a/b (*r* = 0.85081, *P* = 0.00036, Spearman’s correlation test for miR-26a; *r* = 0.79941, *P* = 0.02852, Spearman’s correlation test for miR-26b), *n* = 14. **i** Correlation between 25(OH)D concentrations and miR-26a/b levels in serum from OLP patient (*r* = 0.44655, *P* = 2.86 × 10^−11^, Spearman’s correlation test for miR-26a; *r* = 0.58412, *P* = 2.87 × 10^-11^, Spearman’s correlation test for miR-26b), *n* = 14. *P < 0.05, **P < 0.01, ***P < 0.001 vs. corresponding control. Ctrl control, VDRKO VDR knockout, Pari paricalcitol, VD-D vitamin D deficiency.

To figure out the mechanism of miR-26a/b reduction in OLP, we evaluated their genomic regions of transcription. Gene database of NCBI showed that miR-26a seems to have two genes (*miR-26a-1* and *miR-26a-2*), which are located in their host genes *CTDSPL* and *CTDSP2* respectively, miR-26b gene loci is localized in the introns of its host gene *CTDSP1* and shares the same promoter with it (Fig. 2a). Bioinformatics analysis by UCSC database revealed that promoters of three miR-26a/b genes all contains transcription factor VDR’s binding sites, which are termed as vitamin D receptor element (VDRE) (Fig. 2a and Supplementary Fig. 2a). VDR is a nuclear hormone receptor and embraces a wide range of biological activities, including immune response suppression and apoptosis inhibition^20^. To confirm the bioinformatics data, we designed primers flanking VDR bind sites and performed ChIP assays. As displayed, VDR protein bound to VDRE robustly after VDR plasmids treatment compared with vector control in HOKs (Fig. 2b). VDR expression was chosen as an internal control and highly increased as well (Supplementary Fig. 2b, d). Consistently, VDR plasmids transfection promoted miR-26a/b and their host genes expression (Supplementary Fig. 2c-e), and rescued LPS or activated CD4^+^ T cells-induced miR-26a/b reduction (Fig. 2c, d). Consistently, both mRNA and protein expression of VDR had ~50% decreases in the two cell models (Supplementary Fig. 2f-k).

Vitamin D is reported to bind and activate VDR in cytoplasm and accelerate its nuclear translocation^21^. To verify the functions of vitamin D/VDR signaling in miR-26a/b, we treated HOKs with 1,25-dihydroxyvitamin D (1,25(OH)_2_D_3_), the active form of vitamin D. Accordingly, 1,25(OH)_2_D_3_ mildly reversed miR-26a/b decreases in the two cell models (Supplementary Fig. 2l). However, this protective effects of 1,25(OH)_2_D_3_ was diminished when HOKs were transfected with hVDR-siRNA (Supplementary Fig. 2m, n). Moreover, acetylation of VDR protein occurs at lysine 91 (K91) which is recognized by bromodomain proteins (BRDs, Supplementary Fig. 2o–p), and pharmacological inhibition of BRD9 (iBRD9) is stated to facilitate physiological functions of vitamin D/VDR signaling via promoting VDR acetylation^22^. Indeed, miR-26a/b levels were much higher in the presence of 1,25(OH)_2_D_3_ and iBPD9 compared with either control or 1,25(OH)_2_D_3_ alone (Supplementary Fig. 2q).

In line with the results in vitro, miR-26a/b had ~40% decreases in VDRKO mice (Fig. 2e). VDR deletion was measured by qPCR and western blotting (Supplementary Fig. 2r). In addition, we set up vitamin D (VD) overexpression and deficiency models in wildtype mice, and verified the mRNA and protein levels of VDR as well (Supplementary Fig. 2s–t). miR-26a/b were upregulated in oral keratinocytes of mice with VD treatment, while decreased mildly in VD-deficient mice (Fig. 2f–g). In human samples, VDR levels displayed good positive correlations with miR-26a and miR-26b in biopsies, and so did them in serum (Fig. 2h, i).

### miR26a/b block apoptotic activities in oral keratinocytes

**Fig. 3:**
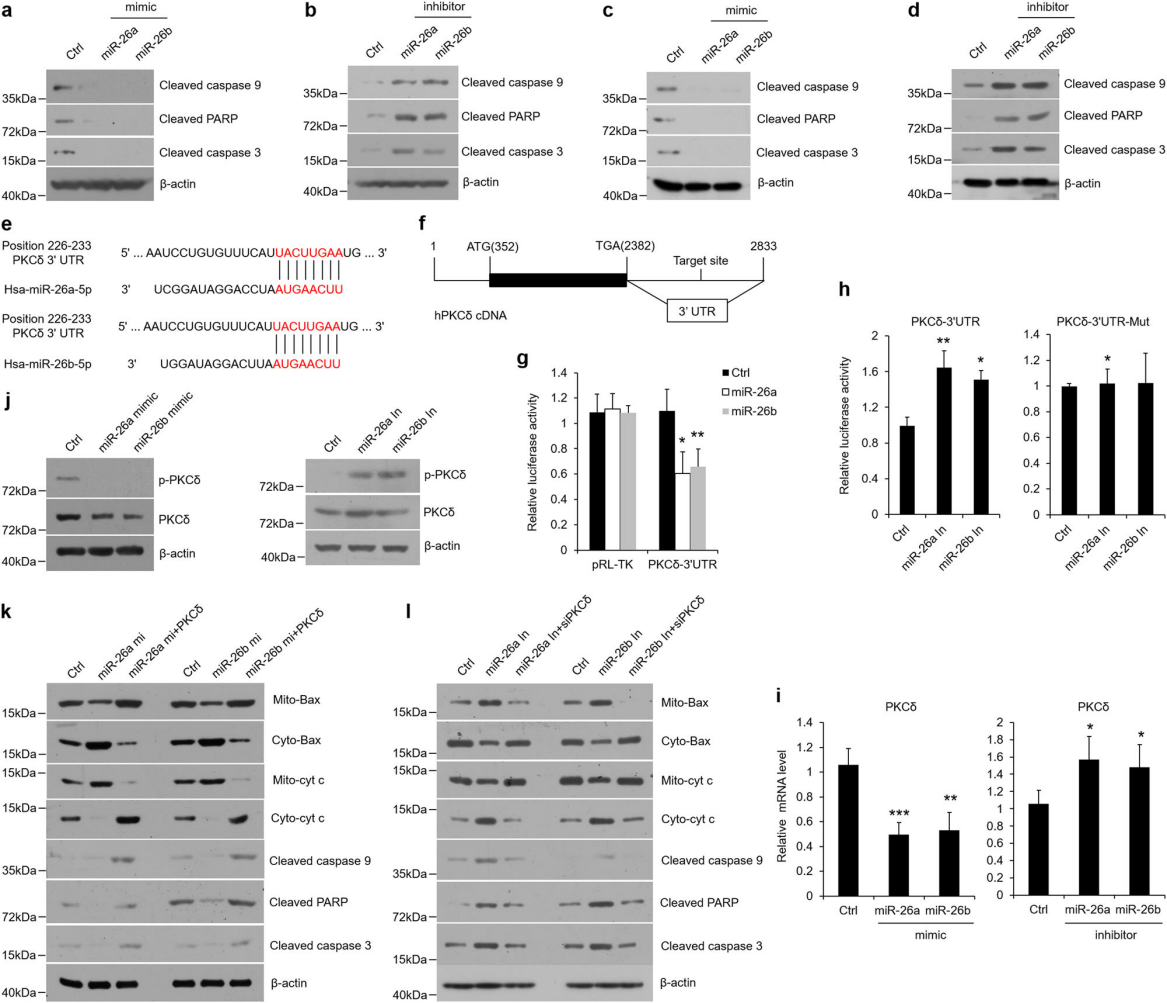
miR-26a/b target PKCδ to inhibit apoptosis in oral keratinocytes. **a**–**d** Western blot showing changes of cleaved caspase 9, cleaved PARP, and cleaved caspase 3 levels in HOKs added with miR-26a/b mimics (**a**) or inhibitors (**b**). **c**-**d** Western blot analysis of cleaved caspase 9, cleaved PARP, and cleaved caspase 3 expression in mouse oral keratinocytes. C57BL/6 mice were subject to miR-26a/b mimics (**c**) or inhibitors (**d**) by tail vein injection. **e** Putative miR-26a/b binding site in the 3′UTR of PKCδ mRNA. **f** Schematic illustration of binding site location in the 3′UTR of hPKCδ cDNA. **g** Luciferase reporter assay showing miR-26a/b target binding site in the 3′UTR of hPKCδ in HOKs which were transfected with Luc-PKCδ-3′UTR or pRL-TK (control) plasmids. Luciferase activity was quantified after 24 h. **h** HOKs were co-transfected with Luc-PKCδ-3′UTR or Luc-PKCδ-3′UTR-Mut and miR-26a/b inhibitors as indicated. **i–j** PKCδ expression and phosphorylation in HOKs with miR-26a/b mimics or inhibitors were measured by real-time PCR (**i**) or western blot (**j**). Western blot showing Bax translocation, cyt c release, and downstream apoptotic factors expression in HOKs during miR-26a/b mimics (**k**) or inhibitors (**l**) treatment with or without PKCδ plasmids or siRNA transfection as indicated. *P < 0.05, **P < 0.01, ***P < 0.001 vs. corresponding control; *n* = 3 for assays in vitro and *n* = 5 for assays in vivo. Ctrl control, Mito mitochondria, Cyto cytoplasm, cyt c cytochrome c, mi mimic, In inhibitor, Mut mutation.

Apoptosis is one of the most common features of OLP^6,23^, thus we evaluated apoptosis-related key factors using miR26a/b mimics or inhibitors in HOKs. As shown, miR26a/b levels were considerably upregulated or down-regulated with miR-26a/b mimic or inhibitor treatment respectively (Supplementary Fig. 3a, b). Meanwhile, suppressions of cleaved caspase 3, cleaved caspase 9, cleaved PARP and caspase 3 activity were observed upon miR26a/b mimics treatment, whereas miR26a/b inhibitors played an opposite role (Fig. 3a, b and Supplementary Fig. 3c, d). Moreover, miR-26a/b mimics attenuated LPS or CD4^+^ T cell secretion-induced apoptosis in HOKs (Supplementary Fig. 3e–g), but the inhibitors of miR-26a/b exacerbated apoptotic activities (Supplementary Fig. 3h–j). To further confirm the roles of miR-26a/b, we injected C57BL/6 mice with miR-26a/b mimics or inhibitors and verified the levels of them in mice oral epithelial cells (Supplementary Fig. 4a, b). Consistently, in mice oral keratinocytes several apoptosis markers were inhibited by miR-26a/b mimic and accelerated by inhibitors (Fig. 3c, d and Supplementary Fig. 4c, d). The same results were also observed in cell models that were established using mice oral keratinocytes (Supplementary Fig. 4e–j), supporting the notion that miR-26a/b suppress apoptosis in oral keratinocytes.

### miR-26a/b target PKCδ to regulate apoptosis in oral epithelial cells

We next searched apoptosis-related potential target genes of miR-26a/b by TargetScan database. Among these hundreds of putative target genes, PKCδ, which is described to induce apoptosis by regulating mitochondrial damage in the cellular context^24^, was predicted to have a binding site of miR-26a/b in the 3′UTR of its mRNAs (Fig. 3e). Further analysis of bioinformatics uncovered that the miR-26a/b binding site in PKCδ was highly conserved across species (Supplementary Fig. 4k). To further confirm it by luciferase reporter assay, we cloned 50 bp nucleotides (2581–2630), which include the miR-26a/b target sequences in 3′UTR fragment of hPKCδ cDNA to a pRL-TK vector (Fig. 3f). Upon miR-26a/b mimics co-transfection, the Luc-PKCδ-3′UTR showed a decrease of luciferase activity (Fig. 3g). miR-26a/b inhibitors had promoted effects on Luc-PKCδ-3′UTR, but not Luc-PKCδ-3′UTR-Mut (Fig. 3h). In consistent, our qPCR and western blot data validated that miR-26a/b regulate PKCδ expression and its phosphorylation (Fig. 3i, j).

These findings above promoted us to assess PKCδ expression in OLP. As shown, PKCδ levels and phospho-PKCδ activities were both enhanced in the two cell models (Supplementary Fig. 5a). During PKCδ activation, Bax translocates into mitochondria and cytochrome *c* releases from the organelles, leading to apoptosis induction^24^. To answer the question that whether PKCδ facilitates apoptotic actions in oral keratinocytes, we constructed PKCδ plasmids and confirmed altered Bax and cytochrome *c* distribution and aggravated apoptosis in HOKs after plasmids transfection (Supplementary Fig. 5b, c). In the following studies, we used two PKCδ inhibitors, rottlerin and δV1–1, to achieve the goals of balancing Bax and cytochrome *c* distributions as well as attenuating apoptosis in cell models (Supplementary Fig. 5d–g). To further test the inhibitory effects of miR-26a/b on PKCδ in OLP, we suppressed or elevated miR-26a/b levels in two cell models. As shown in Supplementary Fig. 5, miR-26a/b mimics compromised PKCδ expression and activities, whereas miR-26a/b inhibitors induced them in HOKs (Supplementary Fig. 5h–k). Next, PKCδ was immunoprecipitated and its tyrosine phosphorylation was analyzed with an antiphosphotyrosine antibody (pY). Our data showed that miR-26a/b regulated OLP-induced tyrosine phosphorylation of PKCδ (Supplementary Fig. 5l, m). The regulatory functions of miR-26a/b were also confirmed in mice oral keratinocytes (Supplementary Fig. 5n–u).

In order to determine whether miR-26a/b target PKCδ to mediate apoptosis, we carried out a couple of rescue experiments in HOKs. First, overexpression of PKCδ by plasmids transfection reversed miR26a/b mimics’ inhibitory functions in apoptosis (Fig. 3k and Supplementary Fig. 5v). Second, upon PKCδ knockdown using siRNA technique (Supplementary Fig. 5x), suppression of PKCδ diminished miR-26a/b inhibitors-induced apoptosis (Fig. 3l and Supplementary Fig. 5w). Collectively, these data suggest that miR-26a/b regulate apoptosis in oral keratinocytes via targeting PKCδ.

### miR-26a/b repress cytokines which are associated with Type 1T helper (Th1) cells in OLP rather than other subsets of Th cells

**Fig. 4:**
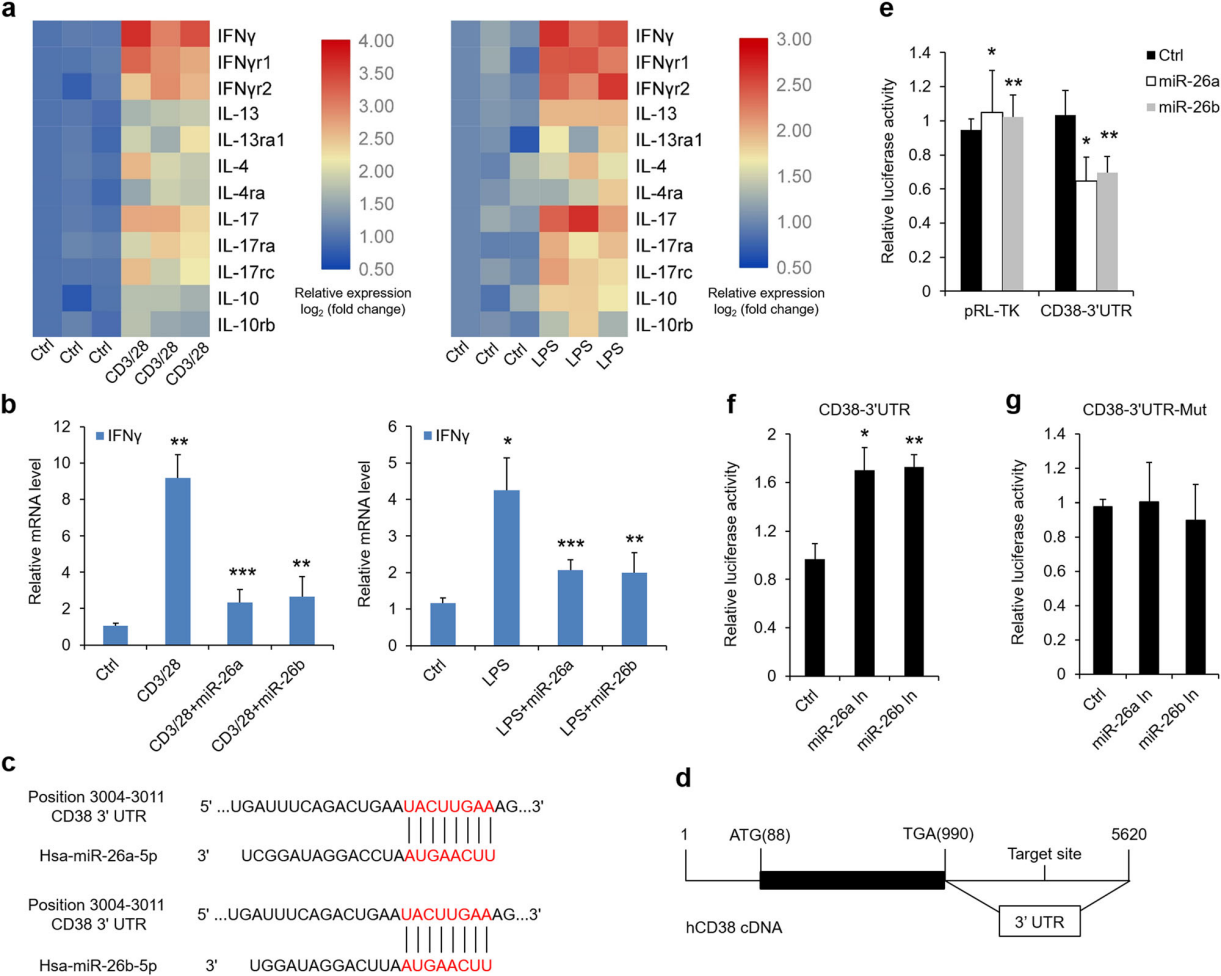
miR-26a/b regulate cytokines and target CD38. **a** Heat map showing alterations of cytokines and corresponding receptors expression in HOKs with activated CD4^+^ T cells or LPS stimulation by real-time PCR. **b** Real-time PCR analysis of relative IFNγ status in activated CD4^+^ T cells or LPS-stimulated HOKs with 36-h miR-26a/b pre-treatment. **c** Putative miR-26a/b binding site in the 3′UTR of CD38 mRNA. **d** Schematic showing binding site location in the 3′UTR of hCD38 cDNA. **e** Luciferase reporter assay showing miR-26a/b target binding site in the 3′UTR of hCD38 in HOKs. f–**g** Luciferase activity quantification in HOKs. Cells were co-transfected with Luc-CD38-3′UTR or Luc-CD38-3′UTR-Mut and miR-26a/b inhibitors as shown. *P < 0.05, **P < 0.01, ***P < 0.001 vs. corresponding control; *n* = 3. Ctrl control, In inhibitor, Mut mutation.

CD4^+^ Th cells seems to be the major lymphocytes in subepithelial and lamina propria areas of OLP^1,2,4^. To investigate it, we evaluated the representative cytokines of Th cell subsets (Th1, Th2, Th17, and Treg cells) as well as their receptors in HOKs. As shown in Fig. 4, the mRNA transcripts of IFNγ, IL-13, IL-4, IL-17, and IL-10 were all considerably increased in cell models, and so were their corresponding receptors (Fig. 4a). To address the question that whether miR-26a/b suppress oral keratinocytes inflammation in OLP, we added miR-26a/b mimics into these cell models. As exhibited, overexpression of miR-26a/b compromised IFNγ levels (Fig. 4b), which is on behalf of Th1 cells, while having no effects on others and all of the receptors (Supplementary Fig. 6a, b). These intriguing data imply miR-26a/b appear to affect Th1-related response, and other Th1 cytokines (TNFα, IL-2, and IL-12) were also tested to confirm this conclusion (Supplementary Fig. 6c, d).

### Cluster of the differentiation (CD38) is a direct target gene of miR-26a/b in oral epithelial cells

To settle the problem regarding how miR-26a/b mediate inflammation, we screened the TargetScan database and found out that CD38, a surface molecule induced by IFNγ^25,26^, is a candidate target gene of miR-26a/b. The bioinformatics analysis indicated there is a highly conserved binding site of miR-26a/b in the 3′UTR of CD38 (Fig. 4c and Supplementary Fig. 6e). In luciferase reporter assay, we cloned 50 bp nucleotides (3978–4027) that contain the miR-26a/b target sequences in 3′UTR fragment of hCD38 cDNA to a pRL-TK vector (Fig. 4d). Upon miR-26a/b mimics co-transfection, the luciferase activities of Luc-3′UTR of CD38 were decreased (Fig. 4e). miR-26a/b inhibitors had promoted effects on Luc-CD38-3′UTR, but not Luc-CD38-3′UTR-Mut (Fig. 4f–g). In agreement, miR-26a/b were confirmed to mediate CD38 protein expression in HOKs (Supplementary Fig. 7e, f) and in mice oral keratinocytes (Supplementary Fig. 7I, j).

### miR-26a/b down-regulate CD38 that is highly increased and contributes to Th1-linked cytokines production in OLP

**Fig. 5:**
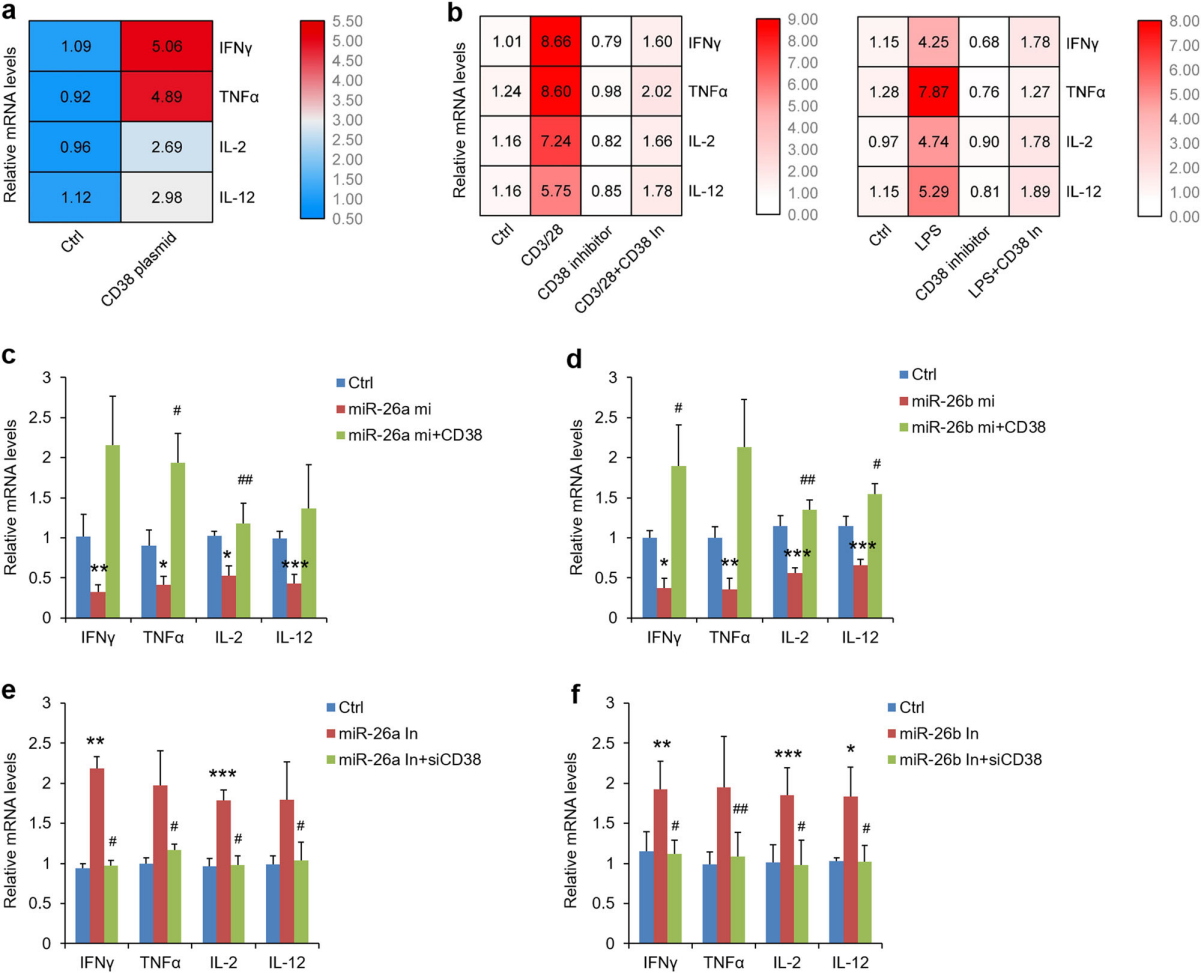
miR-26a/b suppress cytokines via targeting CD38. **a** IFNγ, TNFα, IL-2, and IL-12 levels detected by real-time PCR in HOKs after CD38 plasmids transfection. (**b**) qPCR showing IFNγ, TNFα, IL-2 and IL-12 levels in activated CD4^+^ T cells or LPS-stimulated HOKs, following CD38 inhibitor treatment. Real-time PCR analysis of IFNγ, TNFα, IL-2 and IL-12 levels in HOKs during miR-26a (**c**) or miR-26b (**d**) mimic treatment with or without CD3 plasmids transfection as indicated. Real-time PCR analysis of IFNγ, TNFα, IL-2, and IL-12 levels in HOKs during miR-26a (**e**) or miR-26b (**f**) inhibitor treatment with or without siRNA transfection. *P < 0.05, **P < 0.01, ***P < 0.001 vs. corresponding control; ^#^*P* < 0.05, ^##^*P* < 0.01 vs miR-26a/b mi or In group; *n* = 3. Ctrl control, mi mimic, In inhibitor.

CD38 is strongly upregulated in inflammatory environment in macrophages and epithelial cells^25,26^. In this present study, both mRNA and protein levels of CD38 were increased significantly in cell models (Supplementary Fig. 7a–d). To address the problem that whether CD38 facilitates Th1-related inflammatory actions in oral keratinocytes, we constructed CD38 plasmids and transfected them into HOKs (Supplementary Fig. 7m). Importantly, overexpression of CD38 accelerated IFNγ, TNFα, IL-2, and IL-12 levels while CD38 inhibitors suppressed them that were induced in these models (Fig. 5a, b), providing compelling evidence for our hypothesis. At the same time, the mediation of miR-26a/b on CD38 expression was also confirmed in OLP cell models both in HOKs (Supplementary Fig. 7g–h) and in mice oral keratinocytes (Supplementary Fig. 7 k, l).

### miR-26a/b regulate Th1-correlated cytokines via targeting CD38 in OLP

To test whether miR-26a/b target CD38 to mediate Th1-correlated cytokines, the rescue experiments were also adapted. Overexpression of CD38 enhanced IFNγ, TNFα, IL-2, and IL-12 levels in the presence of miR-26a/b mimics (Fig. 5c, d). However, miR-26a/b inhibitors lost their abilities of inducing cytokines under CD38 knockdown condition (Fig. 5e, f and Supplementary Fig. 7n–p). In short, these findings support the viewpoint that miR-26a/b regulate Th1-correlated cytokines in oral keratinocytes via targeting CD38.

### PKCδ activities and CD38 expression are increased in OLP biopsies

**Fig. 6:**
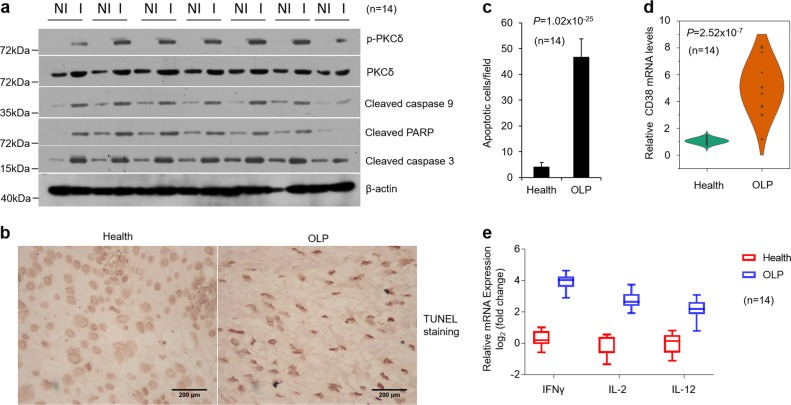
Apoptosis and inflammatory response are enhanced in oral keratinocytes derived from OLP biopsies. **a** Western blot analysis of oral epithelial cells from OLP patients (I) or healthy individuals (NI) with a set of antibodies as indicated. **b** TUNEL staining of biopsies harvested from OLP patients or healthy control. **c** Average TUNEL positive-oral keratinocytes per microscopic field (magnification, ×400), 20 random fields were counted for each group. **d** Violin plot showing CD38 mRNA expression in healthy or OLP oral epitheliums determined by real-time PCR. **e** Real-time PCR analysis of IFNγ, IL-2 and IL-12 mRNA levels in human biopsies. *n* = 14 each group, NI non-inflammation, I inflammation.

We further isolated epithelium from OLP inflammatory biopsies and found PKC activities were enhanced (Fig. 6a and Supplementary Fig. 8a, b). Accordingly, in contrast to healthy controls, increased apoptotic factors were confirmed by western blot and a large number of keratinocytes death were observed by TUNEL staining in diseased tissues (Fig. 6a–c and Supplementary Fig. 8a). In addition, CD38 expression, cytokines of four Th cell subsets and their receptors status were increased as well in lesion samples compared with healthy controls (Fig. 6d, e, Supplementary Fig. 8b, c and Supplementary Fig. 1a).

## Discussion

OLP is one of the most common disorders in oral cavity, with over than 2% of the cases being potential to transform to tumor in clinic^1^. Understanding the molecular causes and identifying the key contributing factors will help us to manage OLP with pharmacological approaches or other strategies. A growing number of evidences demonstrate that miRNA network plays a very important role in this incurable disease^7,10,11^. Investigations of miRNAs functions in OLP appears to be a critical path for OLP exploration. In this study, we have suggested that miR-26a/b is down-regulated in OLP disease, upon reduction, miR-26a/b fail to coordinate programmed cell death and inflammatory cytokines in oral keratinocytes, establishing miR-26a/b as key modulators in OLP.

miR-26 is evolutionarily conserved, but its mediation and effect are a large mystery. Previous studies have demonstrated that miR-26b shows a dramatic decrease in biopsies of OLP patients^27^, implying miR-26 family is involved in OLP development and encouraging us to elucidate the reasons of miR-26 reduction. Consistently, we verified that both miR-26a and miR-26b levels were down-regulated in OLP patients and in cell models. Mechanistically, we found and validated VDR as a critical upstream factor for miR-26a/b induction in HOKs. Although our previous studies have demonstrated that vitamin D/VDR signaling pathway plays a protective role in the microenvironment of oral keratinocytes in OLP^18^, the mechanism of it is not explained completely. This time we provided a positive correlation between vitamin D/VDR and miR-26a/b, which is helpful for better understanding of vitamin D/VDR pathway’s effect on OLP. These results also reveal that vitamin D supplement may be an efficient strategy to increase miR26a/b levels in OLP patients. Since most of our OLP cases encountered in clinic are classified as reticular subtype, it is limited for us to investigate other forms.

We have suggested that apoptosis of keratinocytes in OLP is very common before^6^. Functionally, in the present study, miR-26a/b restricted cell apoptosis in both human and mouse oral keratinocytes by targeting PKCδ. However, other studies note that miR-26a/b enhance apoptosis in various tumor cells^15,28^. The inconsistent discoveries might be due to different cell types used in these studies. PKCδ has emerged as a prominent regulator of apoptosis action by disrupt mitochondrial homeostasis, including Bax translocation into mitochondria and cytochrome *c* release into cytoplasm^24^. Here, we made the novel observation that PKCδ activity was induced in OLP biopsies as well as in cell models. In agreement with other studies, our gain-of-function and loss-of-function assays indicated PKCδ is responsible for the mitochondria damage and downstream apoptotic response in HOKs. Since some attractive investigations have stated that miR-26a/b suppress or enhance apoptotic response by targeting various signaling pathways^28,29^, it is still a mystery that whether miR-26a/b inhibit apoptosis, at least in part, via regulating other pathways in oral keratinocytes. Given that miR-26a/b is possible to enhance apoptosis in tumor cells, it will be very interesting to investigate the roles of miR-26a/b in oral cancer which is transformed from OLP disease. Moreover, the expression of miR-26a/b in oral cancer may be elevated because VDR is reported to be upregulated in oral cancer^30^, but this hypothesis requires further investigations.

In addition to keratinocyte apoptosis, inflammatory response in both epithelial layer and lamina propria is another main feature in the context of OLP. Herein, this study focused on inflammation occurred in epithelium only. We observed severe inflammation activities in OLP samples and cell models in which Th1, Th2, Th17 and Treg-related cytokines as well as their receptors were dramatically increased, consistent with the notion that Th1/Th2 imbalance, Th17 induction and the emergence of Treg cells are all involved into the immunopathological network of OLP^31^. Whether miR-26a/b regulate epithelial inflammation drives us to carry out related experiments. Indeed, we demonstrated the interesting observation that miR-26a/b mimics ameliorated Th1-associated cytokines secretion in cell models except for other kinds of cytokines and all receptors, in parallel with the notion that miR-26 diminished TNFα and NF-κB pathway in alveolar basal epithelial cells^17^. Since most of previous studies have paid more attentions to the inflammatory reaction in the lamina propria of OLP but not epithelial layer^1,31,32^, the function of miR-26a/b in cytokines production of oral keratinocytes provides a new insight into the understanding of OLP development.

**Fig. 7:**
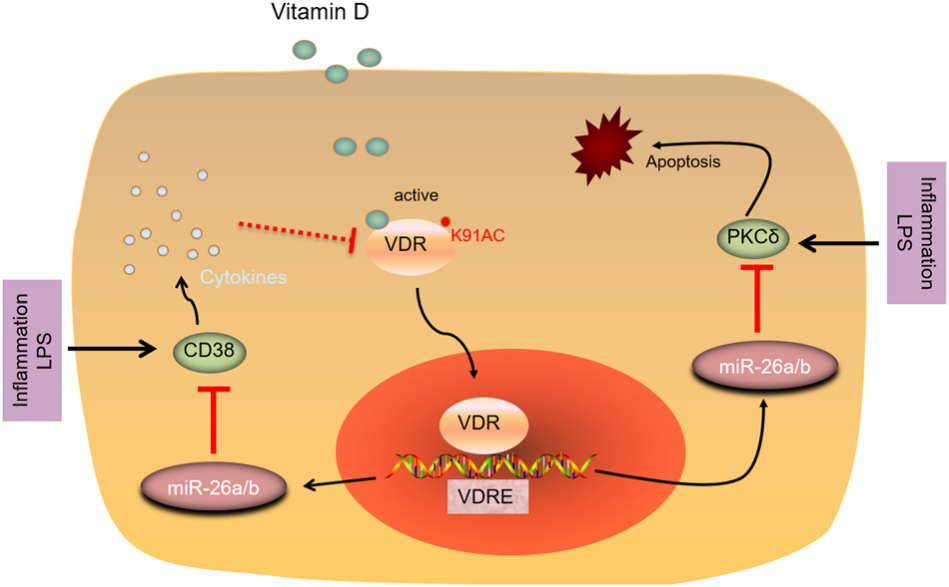
Schematic illustration recapitulates biological process and function of miR-26a/b in oral keratinocytes.

CD38 is an inflammatory marker recognized in macrophages and shows a high increase in the presence of LPS or IFNγ^25^. We employed a variety of approaches to reveal significant up-regulation of CD38 in OLP disease and indicate miR-26a/b fight against inflammation by virtue of targeting CD38. It is attractive that PKCδ inhibitor is demonstrated to suppress CD38 activity^33^, which potentially provides an indirect way for the regulation of miR-26a/b on CD38 expression. To detect the roles of CD38 in inflammation, we transfected CD38 plasmids into HOKs and demonstrated Th1-associated cytokines were induced, whereas CD38 inhibitor blocked them. In this part, one limitation is that the underpinning underlying CD38-mediated regulation of cytokines secretion has yet to be explained, which requires more investigations in the future. In addition, some investigators have pointed out that cytokines such as TNFα damage VDR expression^34^. Thus, cytokines in oral keratinocytes may target VDR degradation as well (Fig. 7), and this feedback regulation is very interesting.

In conclusion, this investigation has suggested miR-26a/b reduction in OLP patients. The decreases of miR-26a/b are due to vitamin D/VDR pathway suppression in the microenvironment of oral keratinocytes, higher levels of these miRNAs would be better for anti-OLP via targeting PKCδ and CD38 respectively (Fig. 7). Discovery of miR-26a/b in oral keratinocytes not only provides novel insights into the comprehension of oral lichen planus but also uncovers new therapeutic approaches for clinic management. Research into lamina propria is not involved in this study, given that, further investigations are needed to guarantee the efficiency of miR-26a/b in OLP treatment.

## Methods and materials

### Human samples collection

OLP patients and healthy participants were recruited in the Stomatological Hospital of Shanxi Medical University. Before medicine treatment, buccal mucosal samples, serum and saliva of participated OLP individuals were obtained for experimental testing. Normal healthy samples were obtained from participants who underwent retained wisdom teeth extraction and had no clinically visible buccal inflammation. Criteria for patient inclusion and identification of OLP were established in terms of the modified World Health Organization diagnostic criteria^35,36^. This project was approved by the Institutional Ethical Committee of Shanxi Medical University and conducted with consent of all individuals. The number of human samples for each group is 14. Clinical parameters of healthy controls were provided in Supplementary Table 1. More OLP patients’ information was provided in previous studies^6^.

### Animal studies

Six to eight-week-old male C57BL/6 mice were selected for this study. VDR^-/-^ mice were generated by reported strategy^37^. To establish vitamin D deficiency model, 3-week-old mice, after weaning, were fed with a vitamin D deficient (vitamin D_3_ < 25IU/kg) or vitamin D normal (vitamin D_3_ = 1000 IU/kg) diet for eight weeks as described before^38^. These mice were placed in a dark room to prevent them from ultraviolet light exposure. In the process of in vivo delivery, wildtype mice were subjected with intravenous injections of 20 mg/kg miRNAs oligonucleotides through tail vein twice one week for 2 weeks with Invivofectamine 2.0 (Invitrogen, cat: 1377901) kit according to the manufacturer’s protocol. The animal part of this investigation was approved by the Institutional Ethical Committee affiliated with Shanxi Medical University. The number of mice for each group is 5. Mice with similar ages were allocated into different groups randomly. The person who conducted the experiments did not know the identity of the specific samples until data were analyzed.

### Oral mucosal epithelial cell isolation

Oral tissues (3 mm × 3 mm) were isolated from human or animal oral bucca, and epithelial layers were separated as demonstrated^6^. Briefly, oral samples were treated with 0.25% dispase II (Sigma-Aldrich) for 12-h digestion, and then epithelial layers were separated by muscle forceps directly.

### Cell culture

Human oral keratinocyte (HOK) cell line (Chinese Beijing North Institution) was placed and cultured in oral keratinocyte medium (OKM, ScienCell, cat: 2611). For mouse oral keratinocyte culture, the separated epitheliums from mouse buccal tissues were cut into small pieces and digested into trypsin (Invitrogen) to get single keratinocytes as described^39^. After wash and resuspension with OKM containing 1% amphotericin B (Sigma-Aldrich), keratinocytes were placed into dishes coated with recombinant human type-1 collagen (Sigma-Aldrich). Cultured medium was added and changed every 2 days. The third-generation keratinocytes were ready for use. For mimicking the microenvironment of OLP in vitro, we established two cells models, respectively. One is that oral keratinocytes were treated with LPS (100 ng/ml, Sigma-Aldrich) for 24 h to mimic the condition of infection-induced OLP, the other one is that supernatants of T cells stimulated with anti-CD3/CD28 (BD Biosciences) were added to cell plates at a 30% final volumetric concentration to mimic the circumstance of inflammation-induced OLP^6^. T cells isolation and stimulation were performed according to previous publication^6^. In the dose-dependent assays, HOKs were treated by LPS (100 ng/ml) or activated CD4^+^ T cells (30% final volumetric concentration) for 0, 4, 8, 16, 24 h independently. In some experiments, after 36-h VDR plasmids transfection (4 µg), 12-h 1,25 VD (20 nM, Sigma-Aldrich) or 12-h PKCδ/CD38 inhibitors (20 nM, MedChemExpress) pre-treatment, HOKs were stimulated with LPS or activated CD4^+^ T cells production for 24 h. Oral keratinocytes were transfected with miR-26a/b mimics or inhibitors (200 nM) for 36 h, followed by LPS or activated CD4^+^ T cells production treatment. All experiments were repeated for three times.

### Plasmids construction

VDR plasmids were kindly provided by Dr. Yanchun Li. For PKCδ or CD38 plasmids construction, the coding sequences of human cDNA were amplified and inserted into pcDNA3.1 vector, according to previous studies^40,41^. To construct luciferase reporter plasmids, 50 bp DNA sequences including predicted target sites of miR-26a/b in the 3′UTR fragment of PKCδ or CD38 cDNA were synthesized and subcloned into pRL-TK vector according to published methods^42^. 50 bp sequences as well as mutations were also used. The inserted sequences are:

AGGGAAATTGTAAATCCTGTGTTTCATTACTTGAATGTAGTTATCTATTG (PKCδ); AGGGAAATTGTAAATCCTGTGTTTCATCGACCTGGTGTAGTTATCTATTG (PKCδ mutation); TTGATTTCAGACTGAATACTTGAAAGGACACACACACACATACGTAAGTG (CD38); TTGATTTCAGACTGAACGACCTGGAGGACACACACACACATACGTAAGTG (CD38 mutation).

### Transfection assay

MicroRNA oligonucleotides (200 nM), siRNA oligonucleotides (40 µM) and plasmids (4 µg) were transfected transiently into oral keratinocytes with the help of Lipofectamin 2000 (Invitrogen). miRNA-26a/b mimics, miR-26a/b inhibitors and the negative controls were purchased from (Thermo Fisher Scientific). Sequences of hVDR-siRNA are 5′-CCCACCUGGCUGAUCU UGUCAGUUA-3′ and 5′-AAUGGCUUCAACCAGCUUAGCAUCC-3′; hPKCδ siRNA sequences are 5′-CCAUGAGUUUAUCGCCACC-3′, hCD38 siRNA sequence are 5′-UUUGGCAGUCUACAUGUCUCAUCUC-3′^18,40,41^.

### Luciferase assay

HOKs were transfected with pRL-TK-3′UTR plasmids or control vectors for 36 h. After transfection, cell lysates were harvested, and luciferase activities were monitored by the Dual Luciferase Reporter Assay System (Promega, cat: E1910) according to manufacturer’s instruction.

### TUNEL and Immunostaining assays

TUNEL staining was adapted as described previously^6^. Oral tissues were fixed by 10% formalin, followed by embedded and cut. Four micrometer sections were then processed according to the protocol of In Situ Cell Death Detection Kit (Roche Life Science, cat: 12156792910). For immunostaining, sections were treated with anti-phospho-PKCδ or anti-CD38 antibodies overnight at cold room, followed by secondary antibodies and diaminobenzidine (DAB) treatments. Slides were observed by a microscope system.

### Elisa

Human blood samples collected on day 1 were placed at 4 °C overnight. After separation on day 2, serum was saved for 25-Hydroxyvitamin D detection using a special kit from Immunodiagnostic Systems following the manual. The concentrations of TNFα in serum and saliva were tested by an Elisa kit from eBioscience. The OD values were measured by a microplate system.

### Western blotting

Western blotting assays were carried out following previous studies^43^. Briefly, cells and tissues were lysed using laemmli buffer and sonicated. Proteins were loaded and separated using 8–12% SDS-PAGE, then transferred to PVDF membranes. After 1-h 5% BSA blocking buffer treatment, membranes were incubated with a series of primary antibodies overnight at 4 °C, followed by 1-hour secondary antibodies incubation at 25 °C. VDR (Cat: sc-13133), CD38 (Cat: sc-374650) and β-actin (Cat: sc-47778) antibodies were from Santa Cruz. CTDSPL (Cat: 17532-1-AP) and CTDSP1 (Cat: 10952-1-AP) antibodies were from Proteintech. CTDSP2 (Cat: ab97463) and PKCδ (Cat: ab182126) antibodies were from Abcam. Phospho-PKCδ (Tyr311, cat: 2055), cleaved caspase 3 (Cat: 9664), cleaved caspase 9 (Cat: 9505, 9509) and cleaved PARP (Cat: 5625, 94885) antibodies were from Cell Signaling. Cytochrome *c* (Cat: MA5-11674) and Bax (Cat: MA5-14003) antibodies were from Invitrogen. Blots were detected using ECL Western Blot Substrate. Βeta-actin was selected as a loading control.

### Coimmunoprecipitation (Co-IP)

Oral keratinocyte lysates were collected using immunoprecipitation lysis buffer and immunoprecipitation was applied with an anti-PKCδ antibody. The resultant precipitates were analyzed by gel electrophoresis.

### Cellular fractionation

For Bax and cytochrome *c* translocations examination, oral keratinocytes were fractionated into cytosolic and membrane-bound organellar fractions by digitonin for immunoblot analysis. Briefly, cells were subjected to a 2-min incubation in isotonic buffer containing 0.05% digitonin at RT. The soluble part of mixture was saved as cytosolic fraction, and then the remaining insoluble materials were dissolved in 2% SDS again for the collection of membrane-bound organellar fraction.

### RNA isolation and real-time PCR

Total RNAs or miRNAs from HOKs or participants were isolated by Trizol reagent (Invitrogen, cat: 15596026) or miRNA isolation Kit (QIAGEN, cat: 217004), respectively. The first strand cDNAs synthesis for mRNA or miRNA were performed with PrimeScript RT Reagent Kit (TaKaRa, cat: RR037B) or a specific miRNA First-strand cDNA Synthesis Kit (Aidlab Biotechnologies, cat: PC4801), respectively. Real-time PCRs were completed with a SYBR Premix Ex Kit (TaKaRa, cat: RR420L) or a miRNA Real-time PCR Assay Kit (Aidlab Biotechnologies, cat: PC4901) accordingly. Relative amount of transcripts of mRNAs was calculated by the 2^-ΔΔCt^ formula. GADPH and U6 were applied as internal control for mRNA testing and miRNA examination, respectively. For circulating miRNA samples, the same amount of exogenous cel-miR-39 was added before miRNA extraction and served as normalization. PCR primers were shown in Supplementary Table 2.

### Caspase-3 activity assay

Caspase-3 activities were measured as previously described^6^. Briefly, oral keratinocytes were lysed using lysis buffer for 10 min on ice. Following centrifugation, supernatants were collected as cell lysate. Caspase-3 activities of lysate were detected by caspase substrate Ac-DEVD-AFC (Bio Vision), and analyzed on a plate reader at Ex360/Em530.

### Chromatin immunoprecipitation (ChIP) assays

ChIP was performed as described previously with some modifications^42,43^. After VDR or control plasmids transfection, HOKs were fixed with 1% formaldehyde and neutralized by glycine. Cell lysates were collected and then sonicated to shear chromatins to get 400–500 bp fragments. Forty micrometers sheared samples were saved as INPUT. The remaining samples were purified with BSA and protein A agarose beads for 2 h. Four micrograms of VDR or control IgG antibodies were added to samples to bind and pull fragmented chromatins down. After overnight incubation, protein A agarose beads were treated to precipitate antibodies. Precipitated and sheared DNA samples were eluted and purified with a set of washes, and then the IP and INPUT samples were quantified using real-time PCR. INPUT considered as internal control. The primers for real-time PCR were provided in Supplementary Table 2.

### Bioinformatics analysis

TargetScan database (http://www.targetscan.org/) was used to predict the targets of miR-26a/b. UCSC database (https://genome.ucsc.edu/cgi-bin/ hgGateway) was chosen for searching the promoter regions for binding sites analysis.

### Statistical analysis

Data were presented as means ± SD. The variances were similar between the groups that were being statistically compared. The data met the assumptions of the tests. Student’s *t* test was chosen for two groups difference analysis and ANOVA were selected for showing the significant difference of multigroup. Dunn’s multiple comparisons was used for one-way ANOVA and Fisher’s least significant difference was for two-way ANOVA. *P* < 0.05 was considered to be significant.

## Supplementary information

supplemental figure legends and tables

supplemental figure 1

supplemental figure 2

supplemental figure 3

supplemental figure 4

supplemental figure 5

supplemental figure 6

supplemental figure 7

supplemental figure 8
