# Supplementary material for: MicroRNA-26a/b have protective roles in oral lichen planus
Source: Cell Death Dis. 2020 Jan 6;11(1):15. doi: 10.1038/s41419-019-2207-8 (PMC6944705; doi:10.1038/s41419-019-2207-8)
Supplement: Supplementary file 1 — supplemental figure legends and tables [file 41419_2019_2207_MOESM1_ESM.docx]

**MicroRNA-26a/b have protective roles in oral lichen planus**

Jie Du^1,9,10*^, Ruifang Gao^1^, Yimei Wang^2^, Tivoli Nguyen^3^, Fang Yang^4^, Yongyan Shi^5^, Tianjing Liu^6^, Wang Liao^7^, Ran Li^1^, Fang Zhang^1^, Xuejun Ge^4^, Bin Zhao^1,8*^

^1^Department of Oral Medicine, Shanxi Medical University School and Hospital of Stomatology, Taiyuan, Shanxi, China.

^2^Department of Endodontics, Shanxi Medical University School and Hospital of Stomatology, Taiyuan, Shanxi, China.

^3^Division of Biological Sciences, Department of Medicine, The University of Chicago, Chicago, Illinois, USA.

^4^Department of Periodontics, Shanxi Medical University School and Hospital of Stomatology, Taiyuan, Shanxi, China.

^5^Department of Pediatrics, Shengjing Hospital of China Medical University, Shenyang, Liaoning, China.

^6^Department of Pediatric Orthopedics, Shengjing Hospital of China Medical University, Shenyang, Liaoning, China.

^7^Department of Cardiology, Hainan General Hospital, Hainan Clinical Medicine Research Institution, Haikou, China.

^8^Department of prosthodontics, Shanxi Medical University School and Hospital of Stomatology, Taiyuan, Shanxi, China.

^9^Institute of Biomedical Research, Shanxi Medical University, Taiyuan, Shanxi, China.

^10^Lead Contact

*Corresponding author: Jie Du, D.D.S., Ph.D., Department of Oral Medicine, Shanxi Medical University School and Hospital of Stomatology, NO. 56 Xinjian South Road, Taiyuan, Shanxi, 030001, China (email: [dj1243@hotmail.com](mailto:dj1243@hotmail.com)), and Bin Zhao, D.D.S., Department of Oral Medicine, Shanxi Medical University School and Hospital of Stomatology, NO. 56 Xinjian South Road, Taiyuan, Shanxi, 030001, China (email: [18636666068@163.com](mailto:18636666068@163.com)).

**Supplemental figure legends**

Supplemental Figure 1. TNFα Changes in human samples, miR-26a/b and TNFα levels in OLP cell models, Related to Figure 1. (a) TNFα mRNA levels of human biopsies tested by real-time PCR, ***P < 0.001; n = 14. (b and c) Elisa analysis showing up-regulated TNFα concentrations in OLP serum (b) or saliva (c), ***P < 0.001; n = 14. (d and e) qPCR showing reduction of miR-26a/b levels in HOKs stimulated by CD3/28-activated CD4^+^ T cells (d) or LPS (e) at different time points, *P < 0.05, **P < 0.01, ***P < 0.001 vs. corresponding control; n = 3. (f and g) qPCR showing the induction of TNFα with a time-dependent activated CD4^+^ T cells (f) or LPS (g) treatment in HOKs, *P < 0.05, **P < 0.01, ***P < 0.001 vs. corresponding control; n = 3. (h and i) Correlation between TNFα and miR-26a/b in HOKs which were stimulated by activated CD4^+^ T cells for 0, 4, 8, 16, 24 hours (r=-0.8053, P=0.0002, Spearman’s correlation test for miR-26a [h]; r=-0.8543, P=0.0001, Spearman’s correlation test for miR-26b [i]), n=3 for each time point. (j and k) Correlation of fold change between TNFα and miR-26a/b in HOKs challenged by LPS for 0, 4, 8, 16, 24 hours (r=-0.7925, P=0.0002, Spearman’s correlation test for miR-26a [j]; r=-0.8586, P=0.0002, Spearman’s correlation test for miR-26b [k]), n=3 for each time point.

Supplemental Figure 2. Vitamin D/VDR signaling affects miR-26a/b expression in HOK, Related to Figure 2. (a) Putative binding sites of VDR (red) in the promoter sequences of human miR-26a/b genes. Initial codon was indicated in bold. (b-c) qPCR showing induced VDR (b) and miR-26a/b (c) expression in HOKs transfected with VDR plasmids. (d-e) Western blot measurements of VDR plasmids-transfected HOKs using an array of antibodies as indicated (d), and corresponding densitometric analysis (e). (f-g) qPCR analysis of VDR mRNA levels in HOKs with activated CD4^+^ T cells (f) or LPS (g) treatment. (h-i) Western blot showing a decrease of VDR protein expression in HOKs with activated CD4^+^ T cells stimulation (h), and densitometric analysis (i). (j-k) Western blot showing reduction of VDR expression in HOKs challenged by LPS (j), and densitometric analysis (k). (l) Real-time PCR detection of miR-26a/b status in HOKs treated with activated CD4^+^ T cells or LPS in the presence or absence of 1,25VD. (m-n) Real-time PCR showing the differential expression of miR-26a/b in hVDR-siRNA and scramble-siRNA (control)-transfected HOKs followed by activated CD4^+^ T cells (m) or LPS (n) treatment with or without 1,25 VD. (o-p) The crystal structure of VDR protein (o) and the amino acid sequences of VDR protein (p), K91 (lysine) was labeled red. (q) qPCR analysis of miR-26a/b in HOKs affected with 1,25VD or iBRD9 (20 nM, R&D) for 12 hours as indicated. (r-t) The alterations of VDR levels in VDRKO mice (r), and in vitamin D-overexpressed (s) or deficient (t) mice determined by qPCR and western blot. *P < 0.05, **P < 0.01, ***P < 0.001 vs. corresponding control; n = 3 for assays in vitro and n = 5 for assays in vivo. Ctrl, control; 1,25VD, 1,25(OH)_2_D_3_; VDRKO, VDR knockout; VD-D, vitamin D-deficiency.

Supplemental Figure 3. miR-26a/b regulate apoptosis in HOKs, Related to Figure 3. (a-b) Real-time PCR detection of miR-26a/b expression in HOKs transfected with miR-26a/b mimics (a) or inhibitors (b). (c-d) HOKs treated with miR-26a/b mimics (c) or inhibitors (d) were measured by caspase 3 activity assay. (e-f) Western blot showing miR-26a/b mimics suppressed cleaved caspase 9, cleaved PARP and cleaved caspase 3 expression in HOKs with active CD4^+^ T cells (e) or LPS (f) challenge, and corresponding densitometric analysis. (g) Active CD4^+^ T cells or LPS-induced caspase 3 activities were dampened by miR-26a/b mimics in HOKs. (h-i) Western blot showing miR-26a/b inhibitors promoted cleaved caspase 9, cleaved PARP and cleaved caspase 3 expression in HOKs with active CD4^+^ T cells (h) or LPS (i) challenge, and corresponding densitometric analysis. (j) Active CD4^+^ T cells or LPS-induced caspase 3 activities were enhanced by miR-26a/b inhibitors in HOKs. *P < 0.05, **P < 0.01, ***P < 0.001 vs. corresponding control; n = 3. Ctrl, control; mi, mimic; In, inhibitor.

Supplemental Figure 4. miR-26a/b mediate apoptosis in mouse oral keratinocytes, Related to Figure 3. (a-b) qPCR analysis of miR-26a/b expression in oral keratinocytes isolated from mice which were delivered with miR-26a/b mimics (a) or inhibitors (b) through tail vein, n = 5. (c-d) Caspase 3 activity determination of oral keratinocytes from miR-26a/b mimics (c) or inhibitors (d)-injected mice, n = 5. (e-f) Western blot showing miR-26a/b mimics attenuated cleaved caspase 9, cleaved PARP and cleaved caspase 3 expression in primary cultured mouse oral keratinocytes treated with active CD4^+^ T cells (e) or LPS (f), and corresponding densitometric analysis, n = 3. (g) Active CD4^+^ T cells or LPS-induced caspase 3 activities were blocked by miR-26a/b mimics in cultured mouse oral keratinocytes, n = 3. (h-i) Western blot showing miR-26a/b inhibitors facilitated cleaved caspase 9, cleaved PARP and cleaved caspase 3 expression in cultured mouse oral keratinocytes with active CD4^+^ T cells (h) or LPS (i) stimulation, and corresponding densitometric analysis, n = 3. (j) Active CD4^+^ T cells or LPS-induced caspase 3 activities were increased in the presence of miR-26a/b inhibitors in cultured mouse oral keratinocytes, n = 3. (k) miR-26a/b binding site is conserved. *P < 0.05, **P < 0.01, ***P < 0.001 vs. corresponding control. Ctrl, control; mi, mimic; In, inhibitor.

Supplemental Figure 5. miR-26a/b affect PKCδ expression and phosphorylation, Related to Figure 3. (a) Western blot showing PKCδ and its phosphorylation expression with either LPS treatment or active CD4^+^ T cells stimulation in HOKs, n = 3. (b) PKCδ overexpression after PKCδ plasmids transfection in HOKs. (C) Western blot showing PKCδ plasmids transfection induced Bax translocation into mitochondria, cytochrome c release, cleaved caspse 9, cleaved PARP and cleaved caspase 3 increases in HOKs, and corresponding densitometric analysis, n = 3. (d-g) active CD4^+^ T cells (d) or LPS (f)-induced Bax translocation, cytochrome c release and apoptosis increase were suppressed by Rottlerin and δV1-1 treatments in HOKs, and corresponding densitometric analysis (e and g), n = 3. (h-i) Western blot showing PKCδ and phospho-PKCδ expression in miR-26a/b mimics-transfected HOKs with active CD4^+^ T cells (h) or LPS (i) treatment, and corresponding densitometric analysis, n = 3. (j and k) Western blot showing PKCδ and phospho-PKCδ levels in miR-26a/b inhibitors-added HOKs with active CD4^+^ T cells (j) or LPS (k) challenge, and corresponding densitometric analysis, n = 3. (l and m) Tyrosine phosphorylation of PKCδ in HOKs during active CD4^+^ T cells and LPS stimulation with miR-26a/b mimics (l) or inhibitors (m) pre-treatment. PKCδ was immunoprecipitated from cell lysates for immunoblot analysis of phosphotyrosine (pY), n = 3. (n-o) PKCδ and its phosphorylation levels were up-regulated in oral keratinocytes harvested from mice which were injected miR-26a/b mimics (n) or inhibitors (o) via tail vein, n = 5. (p-q) PKCδ and phospho-PKCδ expression were suppressed in active CD4^+^ T cells (p) or LPS (q)-stimulated primary cultured mouse oral keratinocytes with 36-hour pre-treatment of miR-26a/b mimics, n = 3. (r-s) PKCδ and phospho-PKCδ expression were increased in active CD4^+^ T cells (r) or LPS (s)-stimulated primary cultured mouse oral keratinocytes with 36-hour pre-treatment of miR-26a/b inhibitors, n = 3. (t-u) Tyrosine phosphorylation of PKCδ in active CD4^+^ T cells and LPS-stimulated primary cultured mouse oral keratinocytes with 36-hour miR-26a/b mimics (t) or inhibitors (u) pre-treatment as indicated, n = 3. (v-w) Caspase 3 activity in HOKs during miR-26a/b mimics (v) or inhibitors (w) treatment with or without PKCδ plasmids or siRNA transfection as indicated, n = 3. (x) PKCδ expression of scramble (control) or PKCδ siRNA-transfected HOKs tested by western blot, n = 3. *P < 0.05, **P < 0.01, ***P < 0.001 vs. corresponding control. Ctrl, control; Mito, mitochondria; Cyto, cytoplasm; cyt c, cytochrome c; mi, mimic; In, inhibitor.

Supplemental Figure 6. Effects of miR-26a/b on cytokines and receptors, Related to Figure 4. (a-b) Quantitative PCR analysis of cytokines and corresponding receptors expression as indicated in activated CD4^+^ T cells (a) or LPS (b)-stimulated HOKs with 36-hour miR-26a/b pre-treatment. (c-d) Quantitative PCR determination of TNFα, IL-2 and IL-12 expression in activated CD4^+^ T cells (c) or LPS (d)-stimulated HOKs with miR-26a/b transfection. (e) miR-26a/b binding site is highly conserved across species. *P < 0.05, **P < 0.01, ***P < 0.001 vs. corresponding control; n = 3. Ctrl, control.

Supplemental Figure 7. miR-26a/b give rise to the inhibition of CD38 expression, Related to Figure 5. (a-b) Relative CD38 mRNA levels in HOKs with activated CD4^+^ T cells (a) or LPS (b) treatment, n = 3. (c-d) Western blot showing CD38 protein expression in HOKs after activated CD4^+^ T cells (c) or LPS (d) stimulation, and corresponding densitometric analysis, n = 3. (e-f) CD38 expression in HOKs transfected with miR-26a/b mimics (e) or inhibitors (f), n = 3. (g-h) Changes of CD38 expression in activated CD4^+^ T cells or LPS-treated HOKs with miR-26a/b mimics (g) or inhibitors (h) pre-treatment, n = 3. (i-j) CD38 levels in mouse oral keratinocytes determined by western blot. These mice were delivered with miR-26a/b mimics (i) or inhibitors (j) by tail vein injection, n = 5. (k-l) Changes of CD38 expression in activated CD4^+^ T cells or LPS-treated primary mouse oral keratinocytes with miR-26a/b mimics (k) or inhibitors (l) pre-treatment, n = 3. (m-n) Western blot showing CD38 expression in HOKs with CD38 plasmids (m) or siRNA (n) transcfection, empty vector or scramble siRNA were used as control. (o-p) Real-time PCR analysis of IFNγ, TNFα, IL-2 and IL-12 levels in HOKs transfected with miR-26a In (o) or miR-26b In (p) in the presence or absence of scramble siRNA as indicated, n = 3. *P < 0.05, **P < 0.01, ***P < 0.001 vs. corresponding control; # P < 0.05, ##P < 0.01 vs miR-26a/b In group. Ctrl, control; mi, mimic; In, inhibitor.

Supplemental Figure 8. Relative expression of apoptosis-associated factors and Th1-related cytokines in human biopsies, Related to Figure 6. (a) Densitometric analysis of phospho-PKCδ, PKCδ, cleaved caspase 9, cleaved PARP and cleaved caspase 3 in oral keratinocytes from human biopsies. (b) Immunostaining examination of the oral tissues from healthy controls and OLP patients, using anti-phospho-PKCδ and anti-CD38 antibodies as indicated (magnification, x200). (c) Split violin plot showing mRNA expression of cytokines and receptors as indicated in healthy or OLP oral epithelia using real-time PCR. *P < 0.05, **P < 0.01, ***P < 0.001 vs. corresponding control, n = 14 each group.

**Supplemental tables**

Supplemental table 1. Clinical parameters of healthy controls recruited in this study

| No. | Age | Sex | Site |
| --- | --- | --- | --- |
| 1 | 40 | Male | Buccal mucosa |
| 2 | 45 | Female | Buccal mucosa |
| 3 | 29 | Female | Buccal mucosa |
| 4 | 20 | Male | Buccal mucosa |
| 5 | 31 | Male | Buccal mucosa |
| 6 | 25 | Female | Buccal mucosa |
| 7 | 27 | Female | Buccal mucosa |
| 8 | 42 | Male | Buccal mucosa |
| 9 | 52 | Male | Buccal mucosa |
| 10 | 26 | Female | Buccal mucosa |
| 11 | 34 | Male | Buccal mucosa |
| 12 | 28 | Female | Buccal mucosa |
| 13 | 43 | Male | Buccal mucosa |
| 14 | 30 | Female | Buccal mucosa |

Supplemental table 2. Primer sequences involved in this work

| Primer name | Forward(5’-3’) | Reverse(5’-3’) |
| --- | --- | --- |
| Hsa-mir-26a | TTCAAGTAATCCAGGATAGGCT |  |
| Hsa-mir-26b | TTCAAGTAATTCAGGATAGGT |  |
| Mmu-mir-26a | TTCAAGTAATCCAGGATAGGCT |  |
| Mmu-mir-26b | TTCAAGTAATTCAGGATAGGT |  |
| U6 | GATGACACGCAAATTCGTGAA |  |
| hIFNγ | TCGGTAACTGACTTGAATGTCCA | TCGCTTCCCTGTTTTAGCTGC |
| hIFNγr1 | AGCGATTCCAGTATCCTCACT | CCAGGCTAAGCACTAGAAAGAGT |
| hIFNγr2 | CTCCTCAGCACCCGAAGATTC | GCCGTGAACCATTTACTGTCG |
| hIL-13 | CCTCATGGCGCTTTTGTTGAC | TCTGGTTCTGGGTGATGTTGA |
| hIL-13ra1 | TGAGTGTCTCTGTTGAAAACCTC | GGGGTACTTCTATTGAACGACGA |
| hIL-4 | CCAACTGCTTCCCCCTCTG | TCTGTTACGGTCAACTCGGTG |
| hIL-4ra | CGTGGTCAGTGCGGATAACTA | TGGTGTGAACTGTCAGGTTTC |
| hIL-17 | TCCCACGAAATCCAGGATGC | GGATGTTCAGGTTGACCATCAC |
| hIL-17ra | GCTTCACCCTGTGGAACGAAT | TATGTGGTGCATGTGCTCAAA |
| hIL-17rc | CTGCCCTTGTGCAGTTTGG | CAGATTCGTACCTCACTCCCTA |
| hIL-10 | GACTTTAAGGGTTACCTGGGTTG | TCACATGCGCCTTGATGTCTG |
| hIL-10rb | TACCACCTCCCGAAAATGTCA | CCCAGTCTGAATGCTCATCTG |
| hTNFα | CCTCTCTCTAATCAGCCCTCTG | GAGGACCTGGGAGTAGATGAG |
| hIL-2 | TACAAGAACCCGAAACTGACTCG | ACATGAAGGTAGTCTCACTGCC |
| hIL-12 | CCTTGCACTTCTGAAGAGATTGA | ACAGGGCCATCATAAAAGAGGT |
| hCD38 | CAACTCTGTCTTGGCGTCAGT | CCCATACACTTTGGCAGTCTACA |
| hVDR | GACTTTGACCGGAACGTGCCC | CATCATGCCGATGTCCACACA |
| hPKCδ | AACCATGAGTTTATCGCCACC | AGCGTTACATTGCCTGCATTT |
| hGADPH | ACCACAGTCCATGCCATCAC | TCCACCACCCTGTTGCTGTA |
| mVDR | GATGCCCACCACAAGACCTA | CGGTTCCATCATGTCCAGTG |
| mGADPH | TGTGTCCGTCGTGGATCTGA | CCTGCTTCACCACCTTCTTGA |
| Hsa-miR-26a-1  VDRE-1 ChIP | AGGAAGCTCCTTTGACGTGG | GGGCTGTGTGGTTCTGGATT |
| Hsa-miR-26a-1  VDRE-2 ChIP | ATGGTAGAACTGCCCATGCC | TAAGGCCCTGTGATGCCAAC |
| Hsa-miR-26a-2  ChIP | GAGACTGGTCTTTGGGAGGC | TGGTCCTGTGTCCAAAGCTC |
| Hsa-miR-26b  ChIP | CTGTCCCTCACACCCCTTTG | GGTGAAGGATGGGCAAGGAG |
| PKCδ plasmid | GGAGCAAGCCAACAGTGGGAC | GTCCCACTGTTGGCTTGCATGTCC |
| CD38 plasmid | ACCCCGCCTGGAGCCCTATG | GCTAAAACAACCACAGCGACTGG |
