# Supplementary figures and images for: MicroRNA-26a/b have protective roles in oral lichen planus
Source: Cell Death Dis. 2020 Jan 6;11(1):15. doi: 10.1038/s41419-019-2207-8 (PMC6944705; doi:10.1038/s41419-019-2207-8)

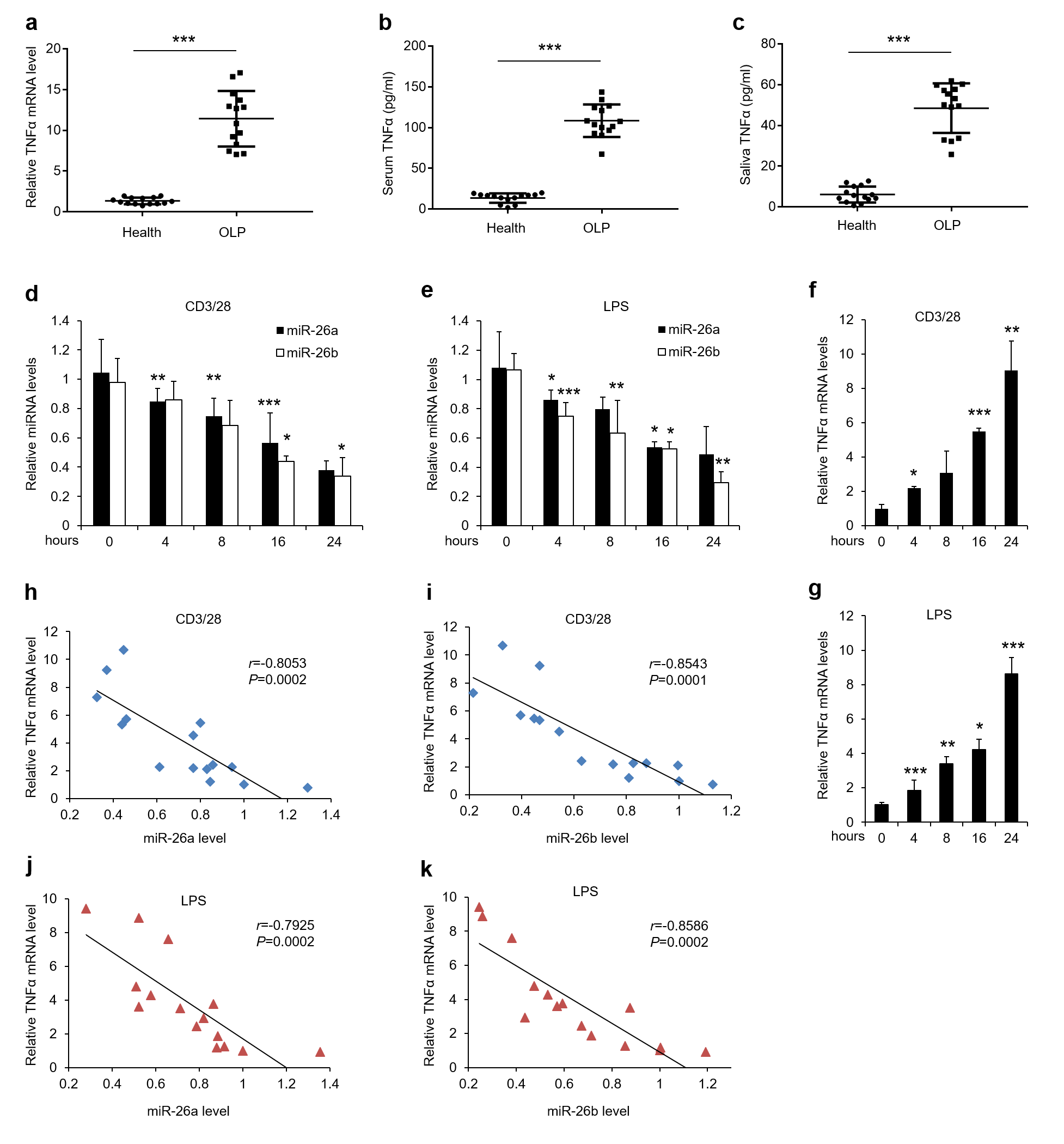

Supplement: Supplementary file 2 — supplemental figure 1 [file 41419_2019_2207_MOESM2_ESM.tif]

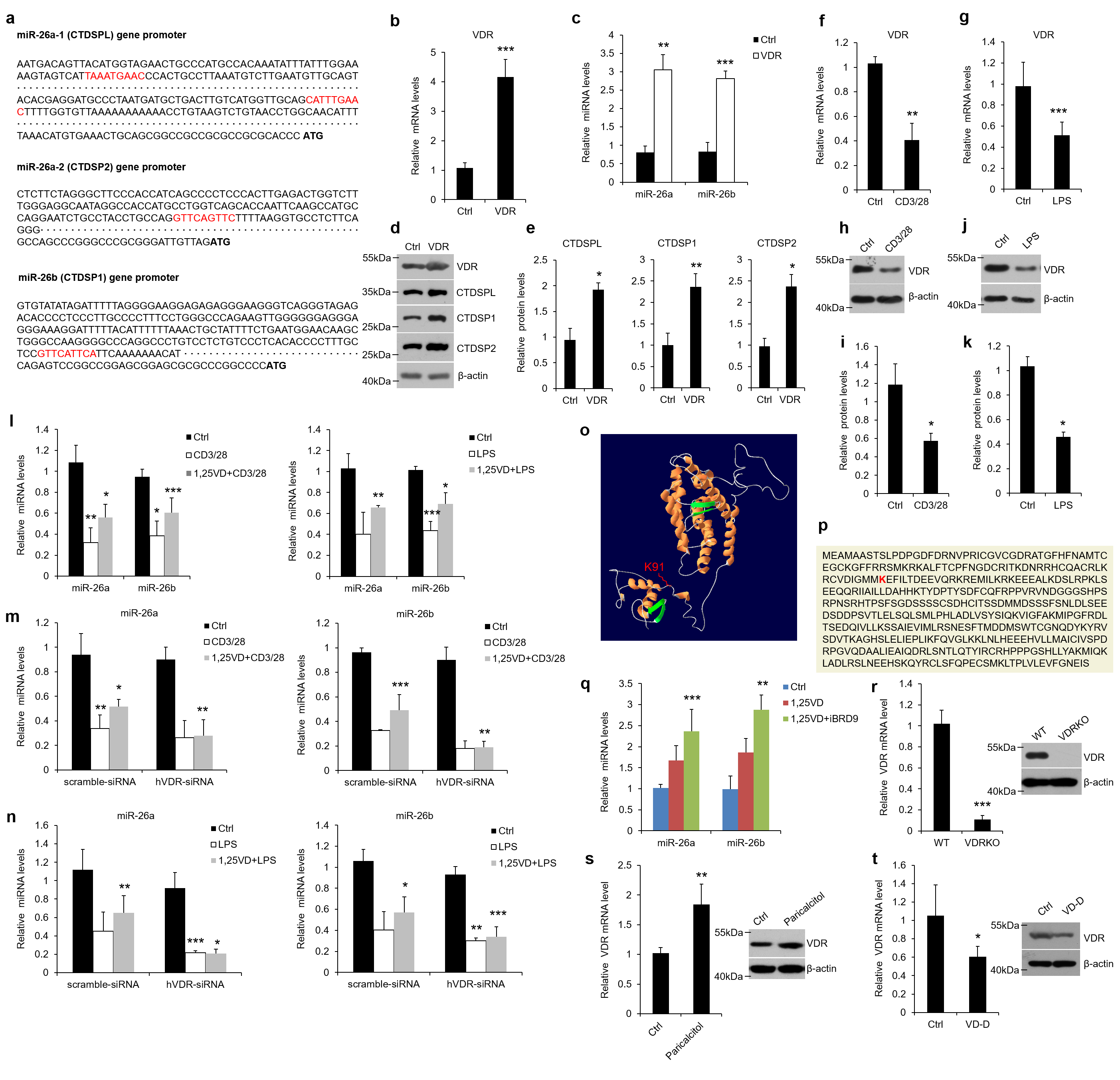

Supplement: Supplementary file 3 — supplemental figure 2 [file 41419_2019_2207_MOESM3_ESM.tif]

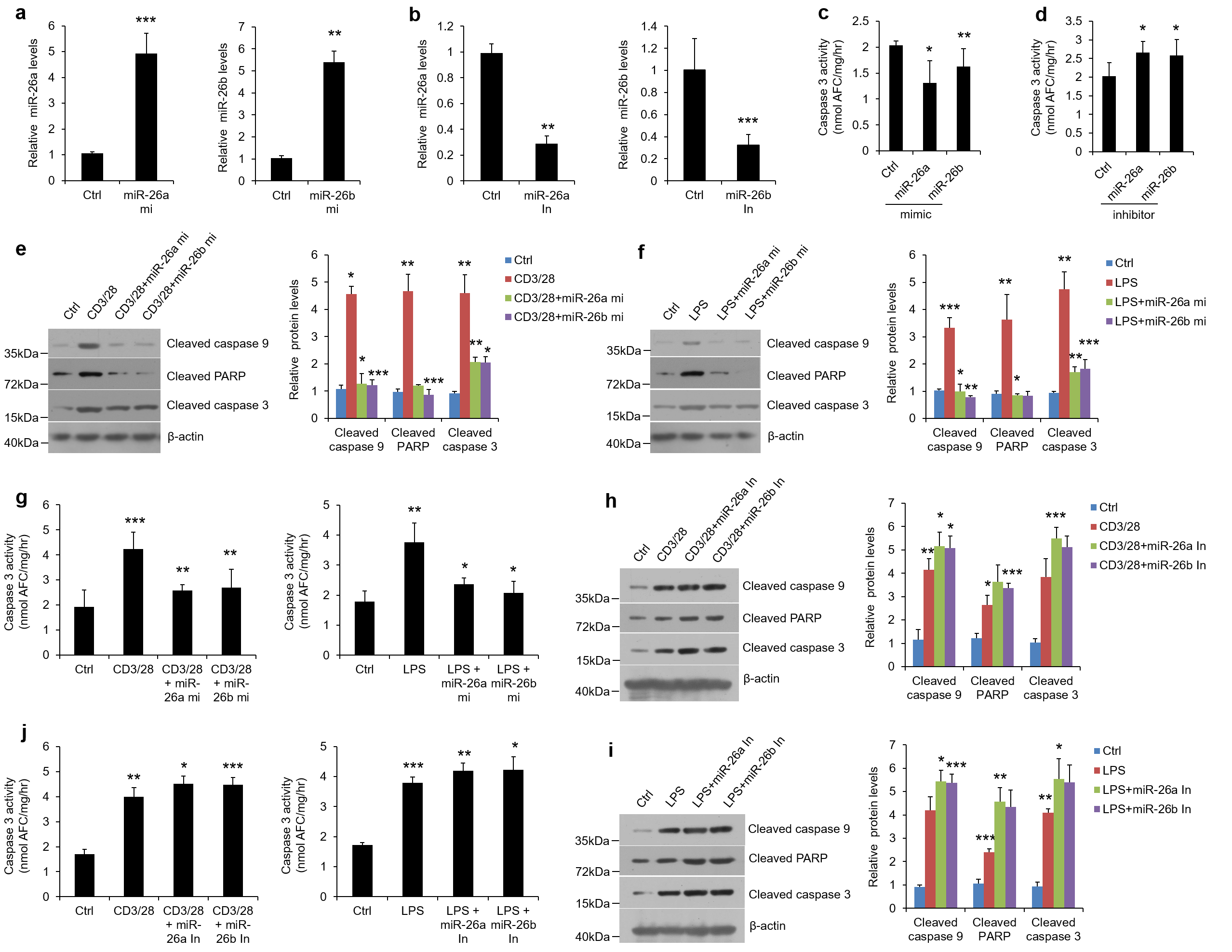

Supplement: Supplementary file 4 — supplemental figure 3 [file 41419_2019_2207_MOESM4_ESM.tif]

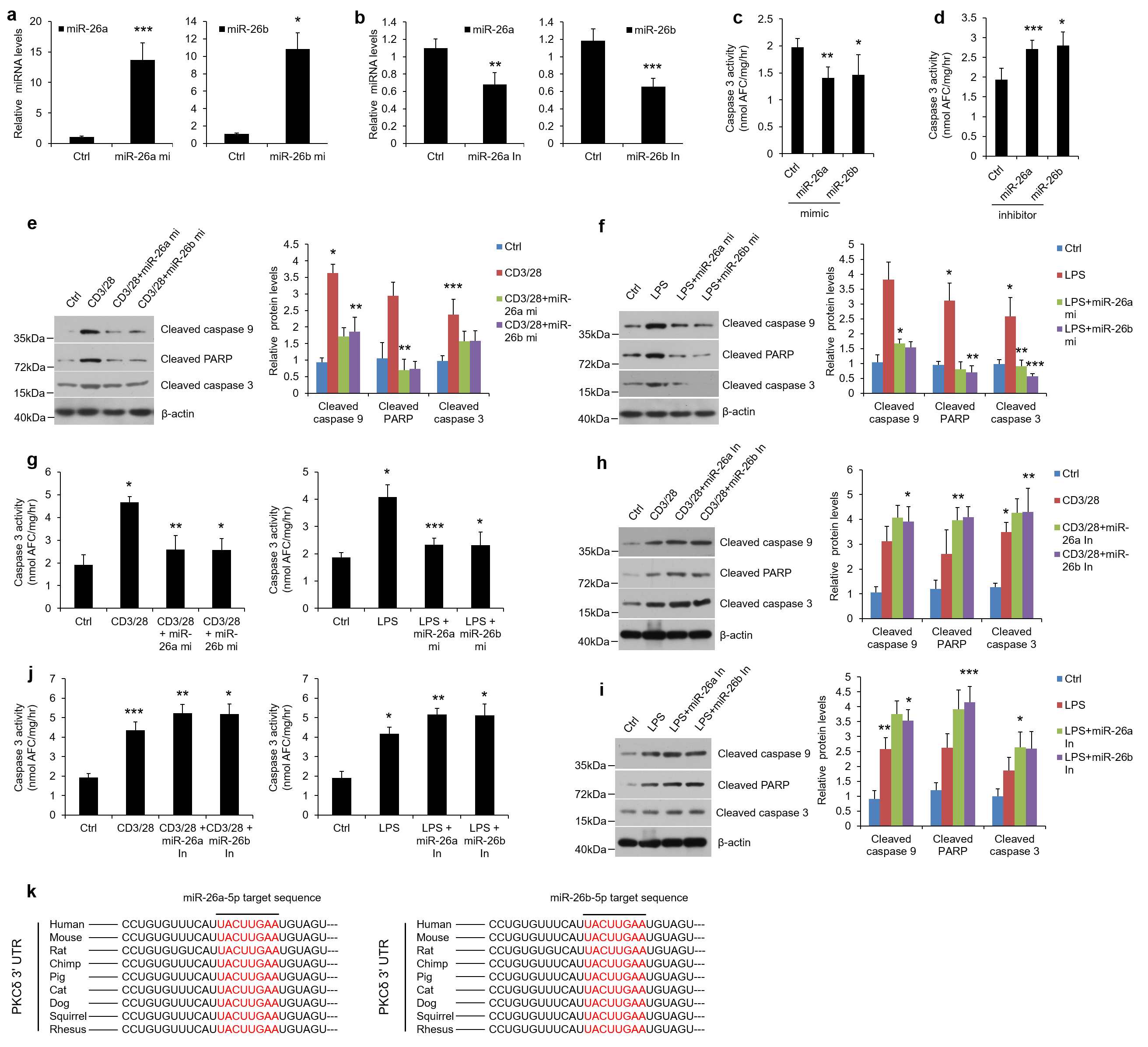

Supplement: Supplementary file 5 — supplemental figure 4 [file 41419_2019_2207_MOESM5_ESM.tif]

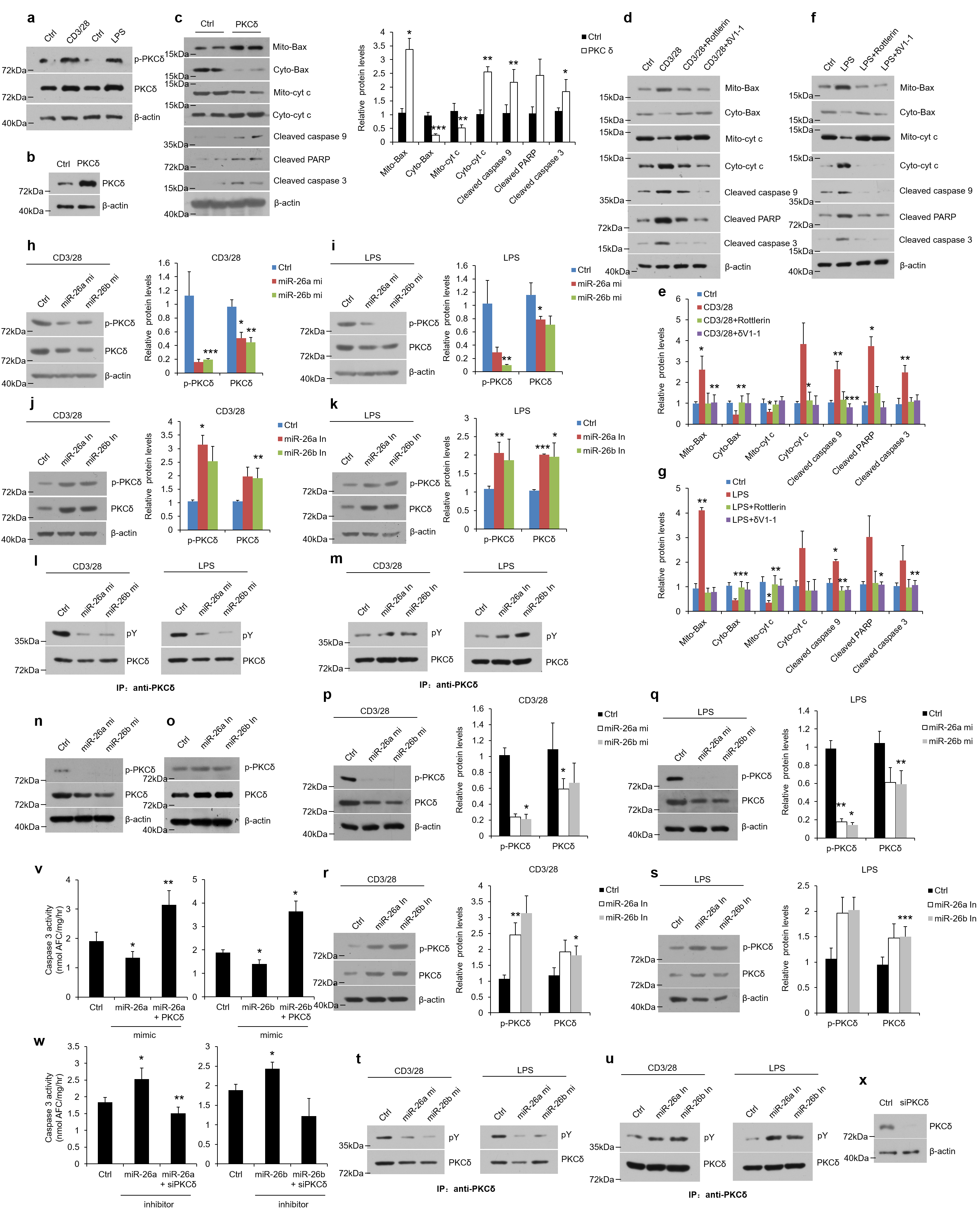

Supplement: Supplementary file 6 — supplemental figure 5 [file 41419_2019_2207_MOESM6_ESM.tif]

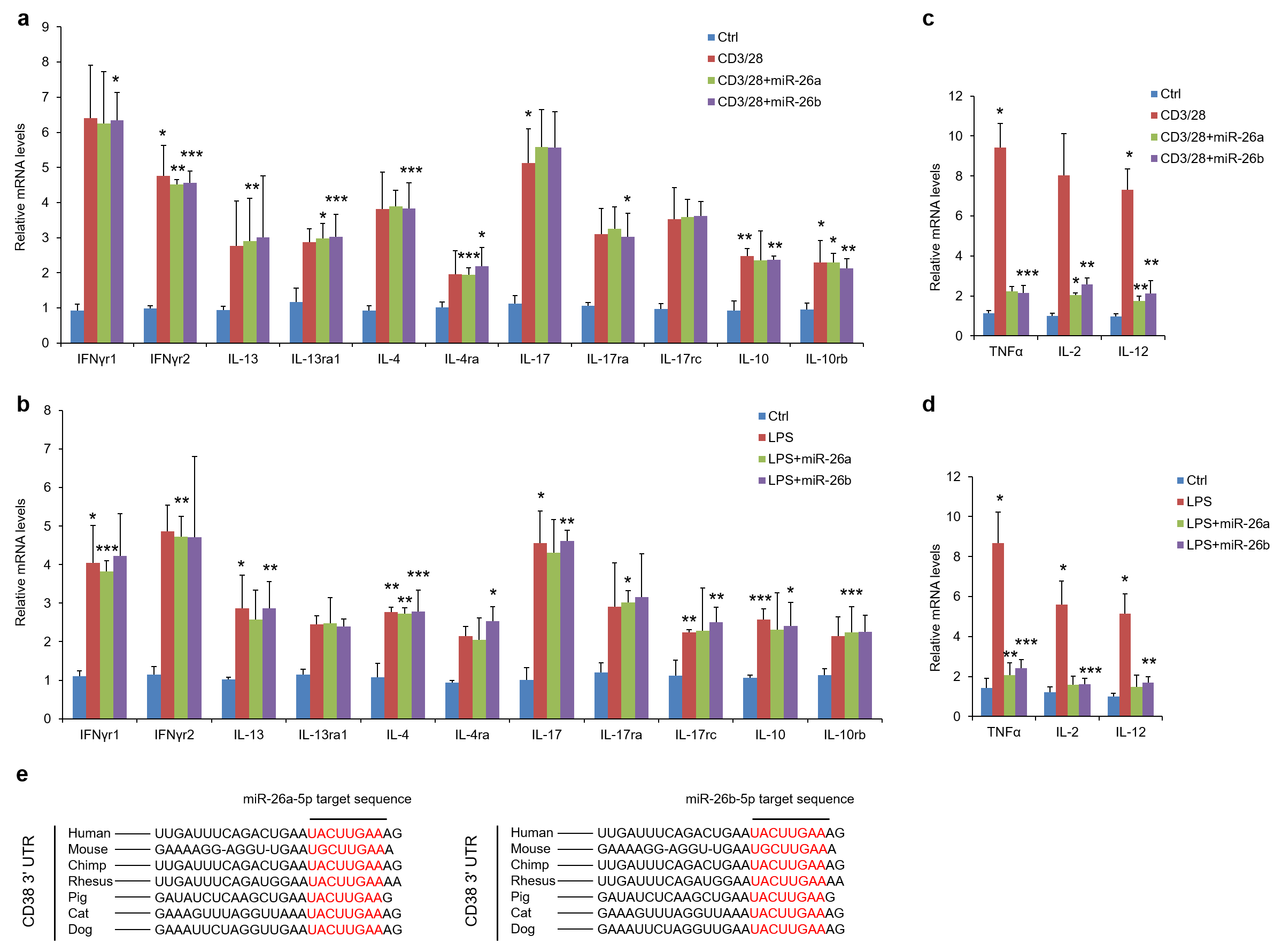

Supplement: Supplementary file 7 — supplemental figure 6 [file 41419_2019_2207_MOESM7_ESM.tif]

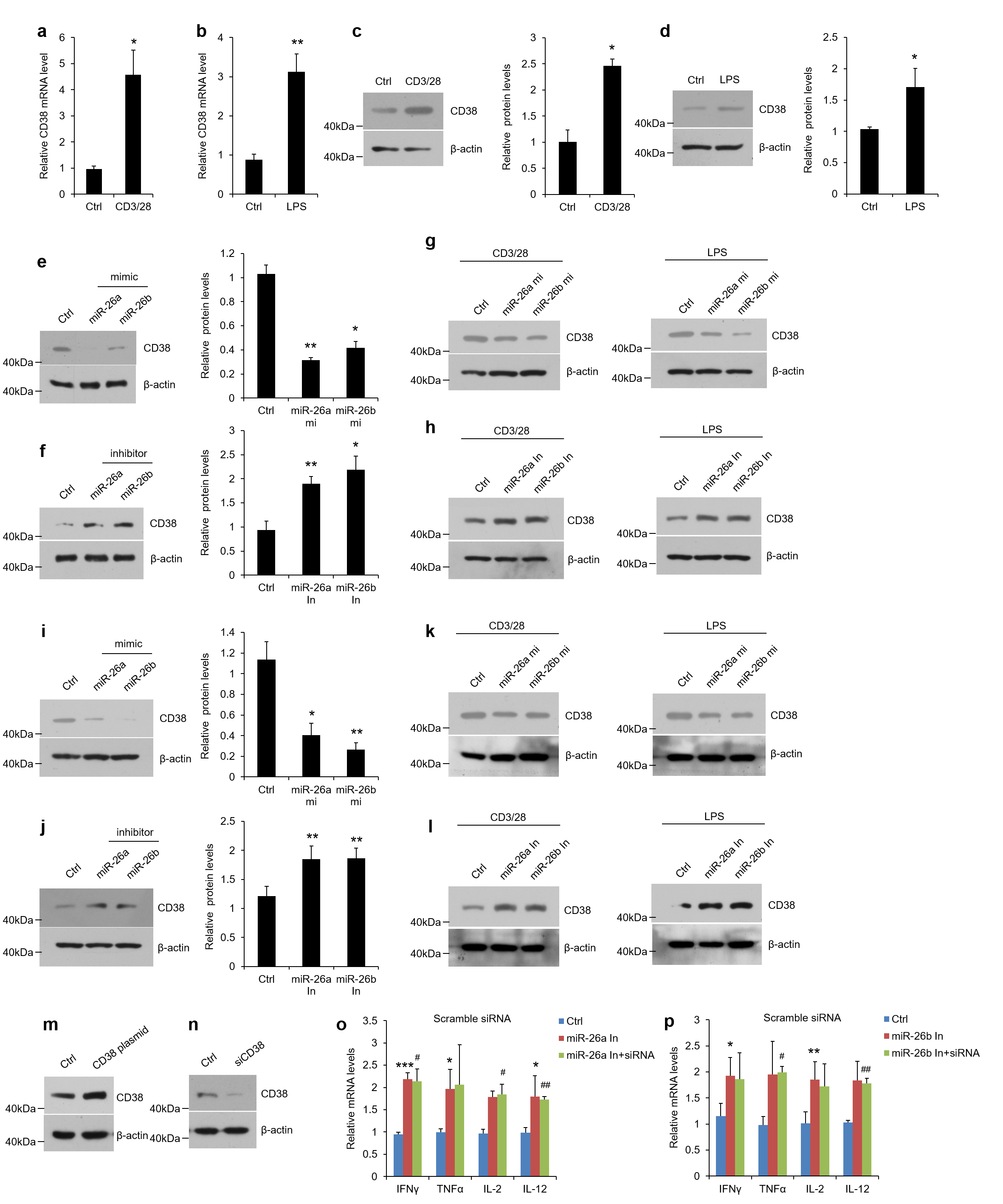

Supplement: Supplementary file 8 — supplemental figure 7 [file 41419_2019_2207_MOESM8_ESM.tif]

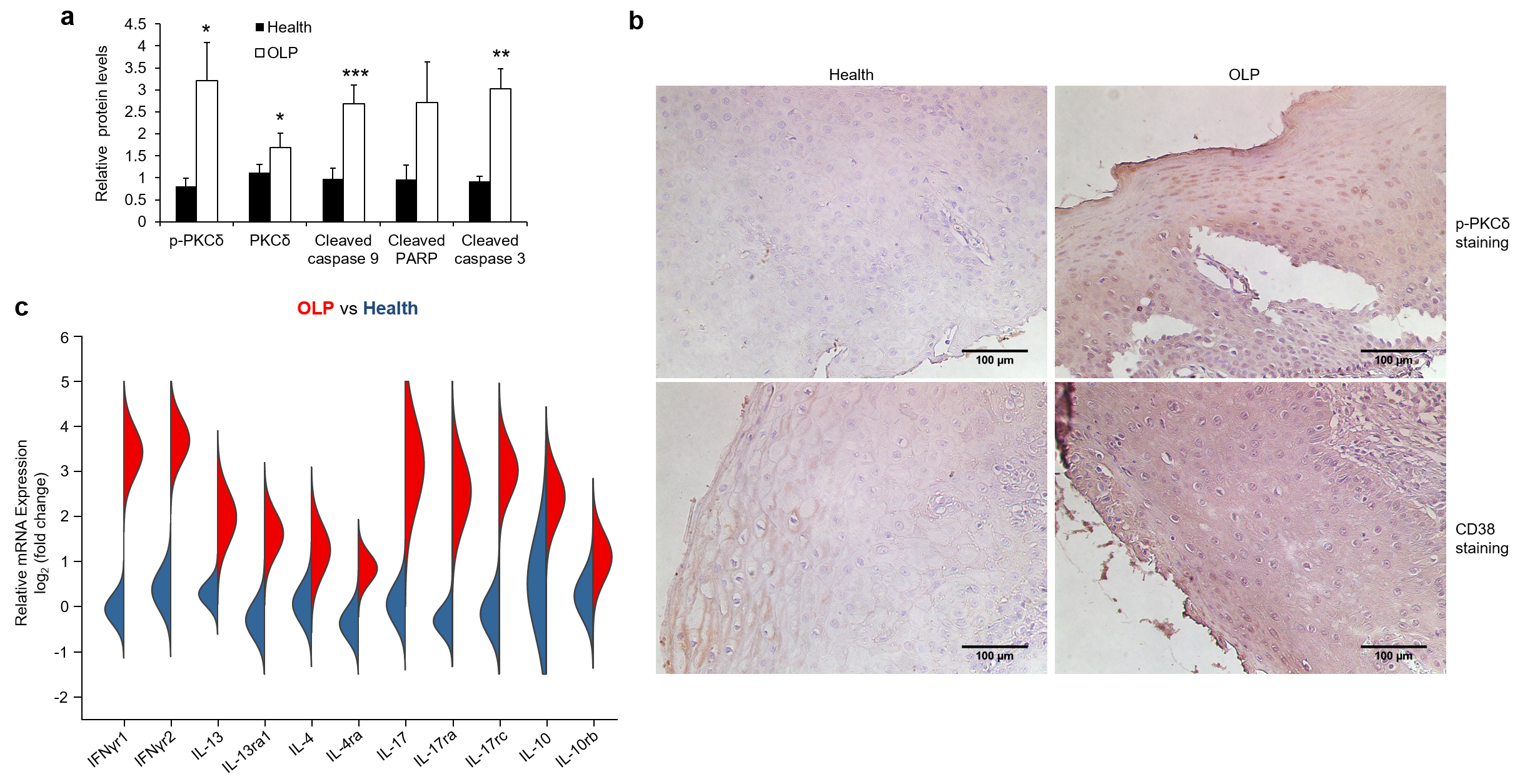

Supplement: Supplementary file 9 — supplemental figure 8 [file 41419_2019_2207_MOESM9_ESM.tif]
